# Pathway and efflux engineering to utilize endogenous high isoprenoid flux in *Azospirillum brasilense* Sp7 for sesquiterpene production

**DOI:** 10.1128/aem.00433-26

**Published:** 2026-05-21

**Authors:** Dattesh Bala Saranga, C. K. Pooja, Amrita Singh, M. V. Adarsh, N. R. Kiran, Chandan U. Gowda, Poonam Kumari, Ankita, Aafreen Zehra, Pranav Murali Sharma, Rudra Prakash Mohanty, V. S. Pragadheesh, Dinesh A. Nagegowda, Mukti N. Mishra

**Affiliations:** 1 Synthetic Biology and Bacterial Functional Genomics Lab, CSIR-Central Institute of Medicinal and Aromatic Plants Research Centre, Bengaluru, India; 2 Molecular Plant Biology and Biotechnology Lab, CSIR-Central Institute of Medicinal and Aromatic Plants, Research Centre, Bengaluru, India; 3 Synthetic Biology Lab, CSIR-Central Institute of Medicinal and Aromatic Plants 30083, Lucknow, India; 4 Analytical Chemistry and Phytochemistry Lab, CSIR-Central Institute of Medicinal and Aromatic Plants, Research Centre, Bengaluru, India; Kyoto University, Kyoto, Japan

**Keywords:** *Azospirillum brasilense*, metabolic engineering, sesquiterpenes, carotenoids

## Abstract

Sesquiterpenes, mainly produced by plants as secondary metabolites, are synthesized from farnesyl pyrophosphate (FPP, an intermediate of the carotenoid biosynthesis pathway) by sesquiterpene synthases (STPSs). The ever-increasing commercial applications have increased the demand for several sesquiterpenes, and the limitations associated with their natural extraction and chemical synthesis have shifted the interest toward heterologous sesquiterpene production using engineered microbes. However, most of the engineered microbes lack the industrial potential yield. Since a high carotenoid yield reflects an equally high flux through FPP, we developed improved carotenoid-producing strains of *Azospirillum brasilense* Sp7 and explored sesquiterpene production using α-humulene and (+)-valencene as two model compounds. A combination of strain construction and media optimization improved the biomass and carotenoid yield >2 and ≈35-fold, respectively. The developed strains were tolerant to the exogenously added sesquiterpenes and organic solvents used for biphasic cultures. Expression of STPSs in the developed strains produced 3.2 ± 0.9 mg/L valencene and 3.25 ± 0.46 mg/L α-humulene, which was improved >10-fold (up to ≈30 mg/L) by co-expressing the rate-limiting enzymes of the 1-deoxy-D-xylulose 5-phosphate pathway. Furthermore, selection and co-expression of the efflux pumps improved the yield >1.5-fold (up to 50 mg/L). We also present evidence that heterologous sesquiterpenes downregulate the isoprenoid/carotenoid pathway in this bacterium. Altogether, our results indicate that *A. brasilense* Sp7 has evolved to carry an endogenous high-flux, squalene-mediated, and dispensable carotenoid pathway, and improvement in the carotenoid productivity improves the sesquiterpene yields up to a certain level. However, sesquiterpene-mediated downregulation of the isoprenoid pathway limits the efficient utilization of its high isoprenoid flux for sesquiterpene production.

**IMPORTANCE:**

To date, several microbes have been engineered, and very few of these are being used for industrial-level sesquiterpene production. However, since most of the engineered microbes lack the industrially potential yield, and since high-yielding microbes are the industrial proprietaries, there is a need for accessible and efficient sesquiterpene-producing microbial hosts, which can be developed by engineering new microbes carrying intrinsic high isoprenoid/carotenoid flux. This study develops *Azospirillum brasilense* strains carrying endogenous, high flux, and dispensable carotenoid pathway and demonstrates the diversion of this flux toward the heterologous sesquiterpenes as well as the bottlenecks limiting the diversion. Although the used engineering approaches could divert only 10% of the total carotenoid flux, it highlights the bottlenecks, which can be addressed to develop this bacterium as an efficient sesquiterpene-producing microbial host. Moreover, for the first time, this work provides an insight into heterologous sesquiterpene-mediated downregulation of the native isoprenoid pathway in bacteria.

## INTRODUCTION

Terpenoids (terpenes or isoprenoids) comprise the largest family of metabolites with >40,000 structurally diverse compounds (1). A relatively small number of terpene compounds (carotenoids, bacterial hopanoids, sterols, ubiquinone, plastoquinone, gibberellins, etc.) are involved in the primary metabolism (2). However, most of the terpenes are not essential for survival and growth, and function as secondary metabolites, which provide survival benefits to the host plants by interacting with their biotic and abiotic environments (3). Terpenes are synthesized from isoprene (C5) building blocks: isopentenyl diphosphate (IPP) and its isomer dimethylallyl diphosphate (DMAPP). Plants use the mevalonate (MEV) pathway in the cytoplasm and the 1-deoxy-D-xylulose 5-phosphate (DXP) pathway in plastids for IPP/DMAPP synthesis. However, with some exceptions, bacteria use the DXP pathway to synthesize IPP/DMAPP (4). IPP and DMAPP are condensed by prenyl transferases in 1:1, 2:1, or 3:1 ratio to produce geranyl pyrophosphate (GPP, C10), farnesyl pyrophosphate (FPP, C15), or geranylgeranyl pyrophosphate (GGPP, C20), respectively. Based on their substrate specificity, terpene synthases utilize GPP, FPP, or GGPP to synthesize mono-, sesqui-, or diterpenes, respectively (3).

Sesquiterpenes, which are synthesized from FPP by the action of sesquiterpene synthases (STPSs), comprise the largest subgroup of terpenoids with >7,000 structurally diverse compounds (1), which serve various ecological roles such as defense against pathogens and herbivores, attracting pollinators, etc. (3). They also possess commercially important properties such as pharmaceuticals, flavoring, fragrance, and biofuel (5). These facts have increased the demand for several sesquiterpenes. For example, the present market volume of (+)-valencene is >15,000 kg/year; ≈10,000 kg is used as such in food-beverage sectors to provide a juicy/citrus impression while ≈5,000 kg is oxidized to produce (+)-nootkatone, which imparts a typical grapefruit aroma at a very low concentration (6). Similarly, the demand for α-humulene and zerumbone (synthesized by α-humulene oxidation) has increased in recent years after realizing their therapeutic potential (7). Although most of the sesquiterpenes are still being extracted from their plant sources, this process leads to poor yields, low purities, and high consumption of raw materials due to their very low concentration in plants. Furthermore, their chemical synthesis is either inherently difficult or economically not feasible. These facts have shifted research interest toward heterologous sesquiterpene production in microbial hosts using metabolic engineering. To date, several microbes have been engineered, and very few of them are being used for industrial production, such as *Rhodobacter sphaeroides*-based valencene production (8). However, since most engineered microbes lack the industrially potential yield (9), and since high-yielding microbes are the industrial proprietaries (8), there is a need for accessible and efficient sesquiterpene-producing microbial hosts. Thus, exploring the sesquiterpene-producing potential of microbes with intrinsic favorable physiological properties may be a promising approach to fulfill the need.

*Azospirillum brasilense* is a plant-growth-promoting and non-photosynthetic bacterium (10), which is generally recognized as safe (11). Earlier, we have reported that *A. brasilense* Sp7 produces a basal level of carotenoids under ambient conditions, but it possesses an endogenous high-flux carotenoid-producing pathway, which is regulated positively by an extracytoplasmic function sigma factor (RpoE1) and negatively by its cognate anti-sigma factor (ChrR1) (12). Recently, we used Car-1 (*chrR1*::mTn*5*), a carotenoid-producing mutant of *A. brasilense* Sp7, to examine heterologous terpene production using geraniol and amorphadiene as a proof of concept (13). Although the reported work demonstrated heterologous terpene production, the issues associated with isoprenoid/carotenoid flux, FPP pool, and extracellular transport of the terpenes have not yet been explored in this bacterium. In this study, we engineer *A. brasilense* Sp7 and optimize its growth conditions to improve the isoprenoid/carotenoid flux, and by using α-humulene and (+)-valencene as two model compounds, we explore the sesquiterpene production in this bacterium with a special emphasis to identify the bottlenecks limiting the diversion of its endogenous high isoprenoid flux toward the sesquiterpenes. This study reveals the intrinsic ability of *A. brasilense* Sp7 to carry a high isoprenoid flux without compromising its growth/biomass and provides evidence that the flux can be utilized for sesquiterpene production by improved/optimized co-expression of STPSs and efflux pumps. By truncating the carotenoid pathway downstream of the FPP, we also provide insight into a tight feedback regulation of the DXP pathway in response to the deletion of the genes involved in the utilization of FPP for carotenoid biosynthesis.

## MATERIALS AND METHODS

### Bacterial strains, growth conditions, plasmids, and chemicals

Table 1 describes the bacterial strains and plasmids used in this study. *A. brasilense* strains were grown at 30°C in Luria-Bertani (LB) or a modified version of minimal malate medium for *A. brasilense* (MMA) (14), which contains 8 g/L of malate. *E. coli* DH5α was grown at 37°C in LB media with appropriate antibiotics to propagate and maintain the plasmids. LB was purchased from Difco (Becton, Dickinson and Company, France), and components of the MMA were from Hi-Media (India). HPLC grade organic reagents, antibiotics, IPTG, α-humulene, and (+)-valencene standard were purchased from Sigma-Aldrich (USA). Restriction enzymes and primers were obtained from NEB (USA) and Sigma-Aldrich (USA), respectively. Nucleotide sequences of the primers used in this study are given in Table S1.

**TABLE 1:** Bacterial strains and plasmids used in this study

Strain or plasmid	Relevant description	Reference or source
*E. coli* strains		
DH5α	Δ*lacU*169 *hsdR*17 *recA1endA*1 *gyrA96 thiLrelA1*	Lab collection
S17-1	*recAthi pro hsdR*^−^ M^+^ RP4:2-Tc:Mu:Km Tn7λpir	(15)
BL21(DE3) LysS	*ompT hsdS* (r_B_^−^ m_B_^−^) *dcm*^*+*^ *endA gal*λ BL21(DE3)	Novagen
*A. brasilense* strains		
Sp7	Wild-type strain	(10)
Car-1	Sp7 *chrR1*::mTn5	(12)
AK03	Sp7 *chrR1*::Km	This work
AK04	Sp7 *ΔchrR1*	This work
AK07	Sp7 *hpnCDE*::Km	This work
AK08	Sp7 *ΔhpnCDE*	This work
AK09	Sp7 *ΔcrtNPOQ*::Km	This work
AK10	Sp7 *ΔcrtNPOQ*	This work
Plasmids		
pKD4	FRT-flanked Km^R^ gene-containing plasmid	(16)
pSUP202	Suicide vector for *A. brasilense*, Ap^R^, Tc^R^, Cm^R^	(15)
pLD132	*flp* containing plasmid; Gm^R^	(17)
pET28-ispA	pET28 containing codon-optimized *E. coli ispA*	(13)
pAK032	IPTG-inducible expression vector; Tc^R^	(13)
pAK052	pET28-*CnVS*	This work
pAK053	pET28-*ZSS1*	This work
pAK060	pET28-*AcHS2*	This work
pAK062	pSUP202-*ΔchrR1*	This work
pAK063	pSUP202-*chrR1*::Km^R^	This work
pAK064	pAK032-*ZSS1*	This work
pAK065	pAK032-*AcHS2*	This work
pAK066	pAK032-*ZSS1-AcHS2*	This work
pAK067	pAK032-*ZSS1-AcHS2-ispA*	This work
pAK068	pAK032-*ZSS1-AcHS2ispA-dxs-idi*	This work
pAK072	pAK032-*CnVS*	This work
pAK073	pAK032-*CnVS-ispA*	This work
pAK075	pAK032-*CnVS-ispA-dxs-idi*	This work
pAK084	pAK032-*flp*	This work
pAK092	pAK032-*rpoE1*	This work
pAK093	pAK032-*chrR1*	This work
pAK105	pAK032-*CnVS-rpoE1*	This work
pAK107	pAK032-*CnVS-ispA-dxs-idi-rpoE1*	This work
pAK108	pAK032-*CnVS-ispA-dxs-idi-AbtolC1*	This work
pAK109	pAK032-*CnVS-ispA-dxs-idi-AbtolC2*	This work
pAK110	pAK032-*ZSS1-AcHS2ispA-dxs-idi-AbtolC1*	This work
pAK111	pAK032-*ZSS1-AcHS2ispA-dxs-idi-AbtolC2*	This work
pAK115	pAK075 + translationally fused *AbtolC1-egfp*	This work
pAK116	pAK075 + translationally fused *AbtolC2-egfp*	This work
pAK135	pSUP202-*ΔhpnCDE*	This work
pAK136	pSUP202-*hpnCDE::Km*	This work
pAK137	pSUP202-*ΔcrtNPOQ*	This work
pAK138	pSUP202-*ΔcrtNPOQ::Km*	This work

### Mutant construction

Mutants were constructed with modifications of the earlier method (12). Briefly, upstream and downstream flanking regions of *chrR1* ORF were amplified using chrR1:AF:EcoRI/chrR1:AR:BglII and chrR1:BF:BglII/chrR1:BR:PstI primer pairs, and cloned between EcoRI/PstI sites pSUP202 (15) to construct pAK062. FRT (Flp recognition target)-flanked Km^R^ gene was amplified from pKD4 (16) using Km:F:BamHI/Km:R:BamHI primers, and cloned into BglII site of the pAK062 to construct pAK063 (pSUP2002-*chrR1*::Km^R^). The pAK063 was conjugatively mobilized into *A. brasilense* Sp7 via *E. coli* S17-1 (15), and exconjugants were selected on MMA plates supplemented with 50 µg/mL of kanamycin (Km). The *chrR1*::Km^R^ mutants were confirmed by PCR and designated AK03. *flp* ORF was amplified from pLD132 (17) using flp:F:PstI/flp:R:KpnI primers and inserted between PstI-KpnI sites of pAK032 (13) to construct pAK084, which was conjugatively mobilized into AK03, and exconjugants were selected on MMA plates supplemented with 10 µg/mL of tetracycline (Tc). The exconjugants were grown in Tc and IPTG-supplemented MMA broth by 3–4 serial passages. Cultures were serially diluted and spread on an MMA plate, and replica-plating was performed to select Km-sensitive colonies. Km^R^ gene excision was validated by PCR, and the *ΔchrR1* mutants were designated AK04. The same procedure was used to construct other mutants used in this study.

### Growth studies and estimation of carotenoid content

Seed cultures of parent (Sp7) and mutant strains (Car-1, AK03, and AK04) were grown in LB or MMA broth, diluted in 100 mL of the respective media up to 0.05 OD_600 nm_, and incubated in an orbital incubator shaker set at 180 rpm and 30°C. Growth was monitored by measuring the OD_600 nm_ after every 4 h until the cultures entered the post-stationary phase. Cells were harvested by centrifuging at 6,000 × *g* for 10 min, washed twice with saline, dried for 60 min at 60°C to remove the extracellular water, and weighed to achieve the biomass. Carotenoids were extracted from the weighed cell pellets by suspending them in methanol and incubating at 4°C. The methanol extraction was repeated until the pellet turned colorless. Absorbance of the methanol extract was measured at 480 nm, and total carotenoids (mg/g biomass) were estimated using the earlier described method (12).

### Estimation of malate consumption

*A. brasilense* strains were grown in MMA, and 1 mL of culture from the zero-hour and stationary-phase (OD_600 nm_ ≈6.5) grown cultures was centrifuged (4,000 × *g*, 10 min, 4°C) to collect the cell-free supernatants. The amount of malate present in the supernatants was estimated by malate dehydrogenase assay using EnzyChrom Malate Assay Kit (BioAssay Systems, USA) according to the manufacturer’s protocol.

### Codon optimization and gene synthesis

Nucleotide sequences of α-humulene synthases of *Zingiber zerumbet* (*ZSS1*) and *Aquilaria crassna* (*AcHS2*), and valencene synthase (*CnVS*) of *Callitropsis nootkatensis* were obtained from GenBank (https://www.ncbi.nlm.nih.gov/genbank/). *ZSS1* (accession no. AB247331.1)*, AcHS2* (accession no. KT893310), and *CnVS* (accession no. JX040471.1) were optimized according to *A. brasilense* codon preferences using the “GenSmart Codon Optimization Tool” available at the GenScript site (https://www.genscript.com/gensmart-free-gene-codon-optimization.html). The optimized sequences were synthesized by GenScript (USA) and cloned in pET28a to construct pAK052 (pET28a-*CnVS*), pAK053 (pET28-*ZSS1*), and pAK60 (pET28-*AcHS2*). Nucleotide sequences of the codon-optimized open reading frames of the STPSs are given in Table S2.

### Protein expression and *in vitro* enzymatic assays

*E. coli* BL21 (DE3) pLysS was transformed by pAK052, pAK053, or pAK060, their cultures in LB media were induced at 0.4 OD_600 nm_ by supplementing with 0.1 mM IPTG, and grown at 18°C with shaking at 180 rpm for 16 h to express the respective proteins. Cells from each culture were harvested and lysed, proteins were extracted from soluble and pellet fractions, and visualized using SDS-PAGE. Enzymatic assays were performed as described earlier (18). Briefly, 250 µL of the soluble fraction and 50 µM FPP were mixed with 500 µL of assay buffer (30 mM HEPES, pH 7.5, 10% [vol/vol] glycerol, 5 mM dithiothreitol [DTT], and 5 mM MgCl^2^). The reaction was immediately overlaid with 200 µL of hexane and incubated for 3 h at 30°C in a test tube. After incubation, the hexane layer was subjected to GC-MS analysis with appropriate standards.

### Construction of valencene and α-humulene biosynthetic operons

The *CnVS* was amplified from pAK052 using CnVS:F:NdeI/CnVS:R:PstI primers (Table S1) and inserted into NdeI-PstI sites of pAK032 (13) to construct pAK072. Codon-optimized *ispA* was amplified from pET28-*ispA* (13) using ispA:F:PstI/ispA:R:XhoI primers and inserted between PstI-XhoI sites of pAK072 to construct pAK073. Dxs:F:XhoI/Dxs:R:KpnI primers were used to amplify *dxs* from *A. brasilense* genomic DNA and cloned into XhoI-KpnI sites of pAK073 to construct pAK074. The *idi* ORF (accession no. AP009048.1) was amplified from *E. coli* genomic DNA using idi:F:NheI/idi:R:KpnI primers and inserted between XbaI-KpnI sites of pAK074 to construct pAK075. idi:R:KpnI primer was designed to introduce an XbaI site upstream to the KpnI site to create XbaI/KpnI sites for insertion of other genes in the pAK075. A similar process was used with appropriate primers to construct plasmids expressing α-humulene biosynthetic operons: pAK064 (pAK032-*ZSS1*), pAK065 (pAK032-*AcHS2*), pAK066 (pAK032-*ZSS1-AcHS2*), pAK067 (pAK032-*ZSS1-AcHS2-ispA*), and pAK068 (pAK032-*ZSS1-AcHS2ispA-dxs-idi*).

### Construction of efflux co-expressing plasmids

*AbtolC1* (AMK58_07740) and *AbtolC2* (AMK58_14050) ORFs were amplified with their native RBSs from *A. brasilense* Sp7 genomic DNA using AbtolC1:F:XbaI/AbtolC1:R:KpnI and AbtolC2:F:XbaI/AbtolC2:R:KpnI primer pairs, respectively, and inserted individually into XbaI-KpnI sites of pAK075 and pAK068 to construct pAK108 (pAK075-*AbtolC1*) and pAK109 (pAK075-*AbtolC2*), pAK110 (pAK068-*AbtolC1*), and pAK110 (pAK068-*AbtolC2*). Translationally fused *AbtolC1-gfp* and *AbtolC2-gfp* ORFs were constructed using standard overlap PCR. Briefly, *AbtolC1* and *gfp* ORFs were amplified using AbtolC1:F:XbaI/AbtolC1(gfp):R and gfp(AbtolC1):F/gfp:R:KpnI primer pairs, respectively. Required nucleotides were included in AbtolC1(gfp):R and gfp (AbtolC1):F to create overlapping regions. These primers were designed to remove the stop and start codons of *AbtolC1* and *gfp*, respectively. The PCR products were used as template with AbtolC1:F:XbaI/gfp:R:KpnI primers to extend the ORFs. *AbtolC2* and *gfp* ORFs were also fused using the same method. The fused *AbtolC1-gfp* and *AbtolC2-gfp* ORFs were inserted into XbaI-KpnI sites of pAK075 to construct pAK115 and pAK116, respectively.

### Production, extraction, and detection of sesquiterpenes

Sesquiterpene production experiments were performed using shake-flask cultures in 100 mL of MMA at 30°C. *A. brasilense* strains harboring different expression constructs were grown up to mid-log phase (3.0–3.5 OD_600nm_), and then IPTG and n-dodecane were added to a final concentration of 1 mM and 10%, respectively. After additional growth of 72 h, the n-dodecane overlay was separated by centrifugation (5,000 × *g*, 10 min, 4°C) and submitted for GC-MS analysis. The cell pellets were dried at 60°C for 1 h and weighed to calculate the sesquiterpene production in terms of per gram biomass. Valencene and α-humulene were identified using GS-MS (Shimadzu Nexis GC-2030 gas chromatograph coupled with GCMS-QP2020 NX mass spectrometer) as reported earlier (13). Sesquiterpenes were quantified by a 7890B gas chromatograph (Agilent Technologies) using the earlier described method (13).

### Data analysis

Each experiment was performed in triplicate at least three times. Statistical analyses were performed using multiple pairwise comparisons using ANOVA followed by Tukey’s post hoc test, and statistically significant differences were indicated either by particular *P*-values or different letters.

### Extraction and detection of squalene and FPP

Cells from 20 mL of MMA-grown stationary-phase cultures of *A. brasilense* strains were harvested (4,000 × *g*, 10 min, 4°C), washed, and resuspended in saline to achieve 5.0 OD_600 nm_. Squalene extraction was performed using the earlier described method (19). Briefly, cells from 5 mL of the adjusted suspensions were harvested, resuspended in 600 µL of freshly prepared alcoholic KOH, and incubated in a boiling water bath for 10 min. After cooling, 600 µL of n-dodecane was added, vortexed, and centrifuged to separate the dodecane layer for GC-MS analysis. Extraction and dephosphorylation of FPP were performed using the earlier described method (20). Briefly, cells from 5 mL of the adjusted suspensions were harvested and resuspended in 2 mL of extraction buffer (butanol/75 mM ammonium hydroxide/ethanol [1:1.25:2.75]), lysed by sonication, incubated at 70°C for 20 min, vortexed, and centrifuged (16,000 × *g*, 15 min, 4°C). The supernatants were evaporated to dryness using a Concentrator plus (Eppendorf, Hamburg, Germany). The dried extracts were resuspended in 0.4 mL of dissolving buffer (1M diethanolamine [pH 9.8], 0.5 mM MgCl_2_) and dephosphorylated by adding 5 U of FastAP alkaline phosphatase (Fermentas, USA). Reaction mixtures were immediately overlaid with 200 µL of n-hexane and incubated at 37°C overnight. Subsequently, samples were vortexed and centrifuged (12,000 × *g*, 5 min, 4°C), and the hexane phase was submitted for GC-MS analysis. A positive control for the dephosphorylation reaction was performed with 5 µL (1 mg/mL) of FPP (Sigma-Aldrich) using the same setup. GC-MS analyses of squalene and farnesol were performed by Shimadzu Nexis GC-2030 gas chromatograph coupled with GCMS-QP2020 NX mass spectrometer using the earlier described methods (19, 20).

## RESULTS

### Construction of strains for improved biomass and carotenoid production

**Fig 1:**
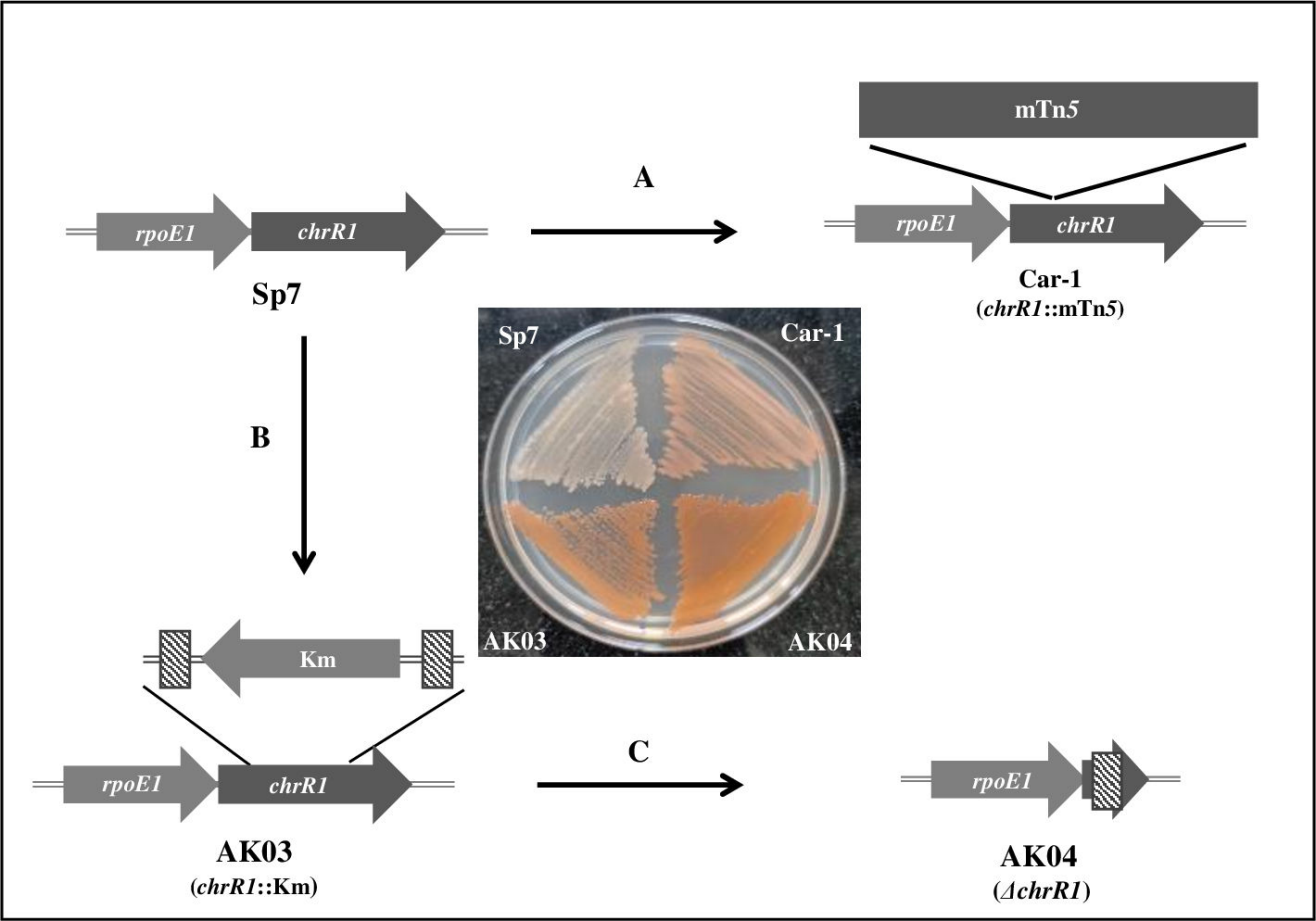
Anti-sigma (*chrR1*) mutants constructed from *Azospirillum brasilense* Sp7 using mTn*5* mutagenesis (**A**) and Km^R^ cassette-mediated replacement (**B**) followed by FLP-mediated excision of the cassette (**C**). Car-1 was made in our earlier reported work (11). AK03 and AK04 are created in this study. Horizontal thick arrows represent the relative positions and transcriptional orientations of *rpoE1* (locus tag, AMK58_01350)*, chrR1* (AMK58_01345), or Km^R^ cassette. The *rpoE1-chrR1* loci of the corresponding strains are schematically shown to depict the genotypic differences in Car-1, AK03, and AK04. Hatched rectangles indicate the Flp recognition targets (FRT) present in the Km^R^ cassette (AK03) and left as a scar in AK04 after excision of the cassette. The culture plate in the center shows the colonies of Sp7, Car-1, AK03, and AK04 on a Luria Bertani agar plate.

**Fig 2:**
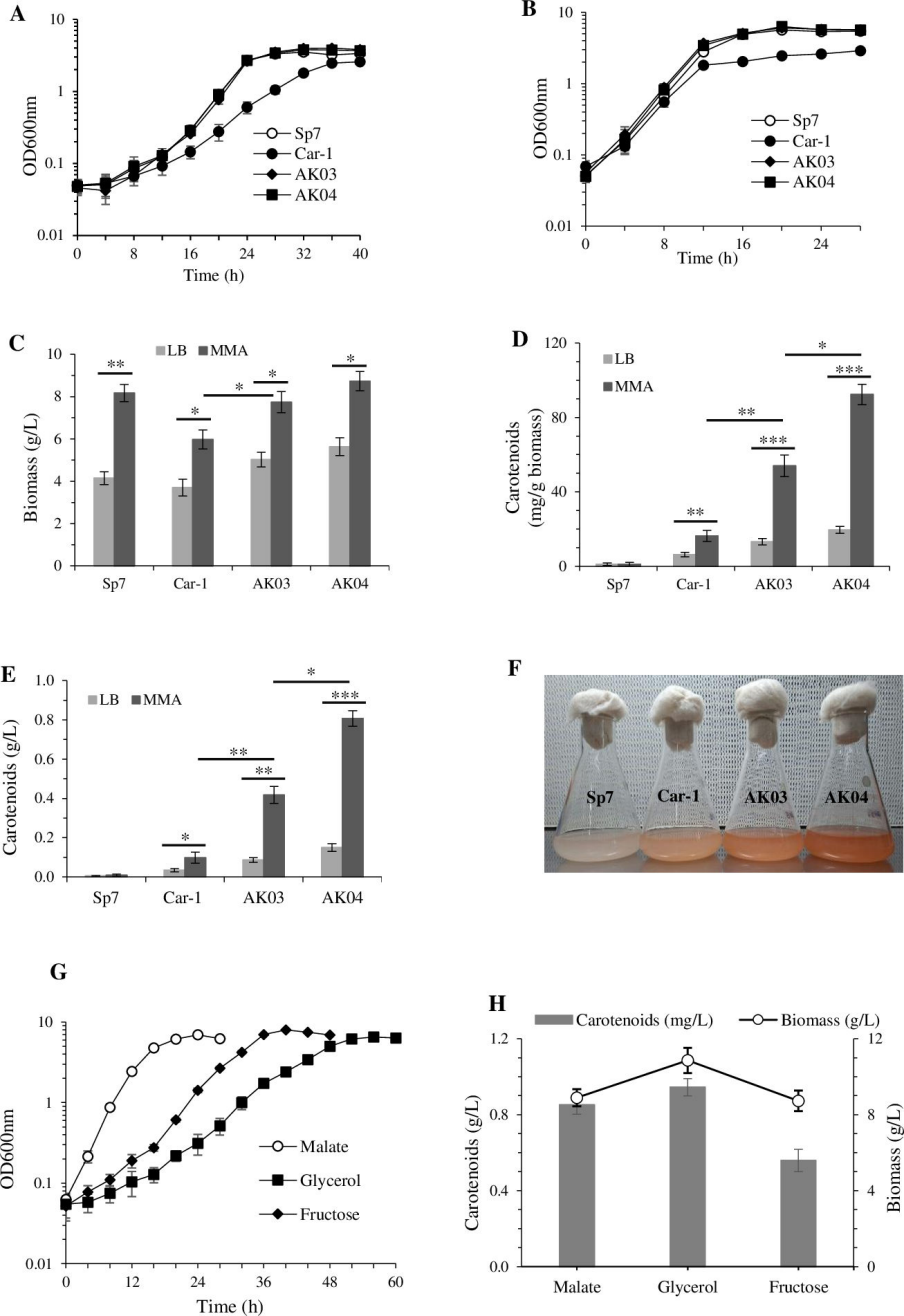
Growth curve of *Azospirillum brasilense* Sp7 (parent) and its three anti-sigma (*chrR1*) mutants in LB (**A**) and MMA (**B**): Car-1 (*chrR1*::mTn*5*), AK03 (*chrR1*::Km^R^), and AK04 (*ΔchrR1*). Growth studies were performed in 100 mL medium in triplicate on three different occasions, and growth was monitored by measuring the OD_600 nm_ after every 4 h. Comparison of biomass (**C**), and carotenoid yields in mg/g biomass (**D**) and g/L (**E**) in MMA and LB. (**F**) Image of MMA-grown culture flasks of Sp7 and the three mutants showing differences in their carotenoid levels. Growth curve (**G**) and biomass/carotenoids yield (**H**) achieved by AK04 using different carbon sources. Each experiment was performed in triplicate on three different occasions. Each data point (**A, B, and G**) and bar (**C, D, E, and H**) shows the mean and standard deviation of values obtained from the triplicate experiments. One, two, and three asterisks indicate the *P*-values <0.05, <0.01, and <0.005, respectively. Wherever not indicated, *P*-values <0.05 were considered for significant differences.

Although carotenoid production in Car-1 (*chrR1*::mTn*5*), which is an anti-sigma factor (ChrR1) mutant of *A. brasilense* Sp7 (Fig. 1), has been reported (12), neither the carotenoid yield nor the effect of constitutive carotenoid production on the growth/biomass has yet been evaluated. Furthermore, the mTn*5*-mediated polar effect on growth/biomass has also not yet been examined. Since these parameters affect the isoprenoid yield, we compared the growth rate of Car-1 with the parent (Sp7) in LB medium, which revealed that Car-1 grows slower than Sp7 (Fig. 2A). We hypothesized that either the mTn5-mediated polar effect or constitutive high-level carotenoid production in Car-1 might be a reason for the growth defect. To examine this, we constructed marked (*chrR1*::Km^R^) and unmarked (*ΔchrR1*) *chrR1* mutants of Sp7 using homologous and FLP/FRT recombination systems (Fig. 1) and compared the growth parameters and carotenoid yield of the mutants with Sp7 and Car-1. Since the FLP/FRT recombination system has not yet been used in *A. brasilense*, we constructed the *chrR1*-disruption plasmid pAK063 (pSUP202-*chrR1*::Km^R^) using the FRT-flanked Km^R^ cassette (15) and an FLP recombinase-expressing plasmid pAK084 (pAK032-*flp*). The pAK063 was used to construct *chrR1*::Km^R^ (AK03) from Sp7 by homologous recombination, while pAK084-borne FLP expression was used to excise the Km^R^ cassette from the AK03 genome to generate the *ΔchrR1* mutant (AK04) (Fig. 1). Visual inspection revealed that AK03 and AK04 produced more intense red colonies than the Car-1 (Fig. 1), and pAK093-borne *chrR1* expression abolished the red color from their colonies (Fig. S1A), indicating complete complementation. Comparative growth analysis revealed that the growth patterns of AK03 and AK04 were similar to that of the Sp7 (Fig. 2A), and AK03/AK04 produced almost equal biomass, which was higher than the Sp7 and Car-1 (Fig. 2C). Interestingly, the carotenoid yields (mg/g biomass) increased ≈2-fold in AK03 and >3-fold in AK04 in comparison to the Car-1 (Fig. 2D). These results not only proved our hypothesis about the mTn5-mediated negative polar effects on growth and carotenoid yield in Car-1 but also suggested that *A. brasilense* has an ability to carry high carotenoid flux without compromising the growth rate and biomass yield.

### Selection of efficient growth media to improve biomass/carotenoid yield

To identify efficient media, we compared the productivity in rich (LB) and minimal medium (MMA) containing 60 mM malate (≈8.0 g/L) as a sole carbon source. It is obvious from Fig. 2A and B that (i) each strain has a smaller lag-phase in MMA than that in the LB and (iii) AK03 and AK04 follow a growth pattern similar to Sp7, while Car-1 follows a defective growth pattern. The achieved biomass by each strain was >1.5-fold higher in MMA than their respective biomasses in LB (Fig. 2C). Interestingly, the carotenoid productivity (mg/g biomass) in MMA increased ≈3-fold in Car-1, >4-fold in AK03, and ≈5-fold in AK04 in comparison to their respective yields in LB (Fig. 2D). Furthermore, the total carotenoid yield (g/L) in MMA increased >3fold in Car-1 (0.098 ± 0.028), >4-fold in AK03 (0.42 ± 0.043), and ≈8-fold in AK04 (0.81 ± 0.045) in comparison to their respective yields in LB (Fig. 2E). Difference in the carotenoid contents of the strains could be observed by visual inspection of their MMA-grown stationary phase culture flasks (Fig. 2F). These results show that MMA is more efficient than LB for biomass and carotenoid production, and the combination of mutant construction and media optimization improved the biomass approximately >2-fold (from 3.68 ± 0.41 g/L by Car-1 in LB to 8.73 ± 0.45 g/L by AK04 in MMA), and the total carotenoid yield ≈35-fold (from 0.023 ± 0.008 g/L by Car-1 in LB to 0.81 ± 0.045 g/L by AK04 in MMA).

The higher productivity in MMA than in the rich medium prompted us to examine the productivity with other carbon sources. Because of its glucose utilization inability, other carbon sources have been identified for *A. brasilense* (21). However, their efficiencies have not been compared. Based on earlier reports, fructose and glycerol were selected because they are less oxidized carbon sources than malate. The selected carbon sources were used (8.0 g/L) separately in place of malate. Comparative analysis revealed that (i) AK04 grows slower with fructose and the slowest with glycerol (Fig. 2G); (ii) an almost equal biomass (≈8.5 g/L) was achieved with malate or fructose while slightly higher (10.80±0.54 g/L) with glycerol (Fig. 2H); and (iii) carotenoid yield (g/L) in glycerol was close to that of malate while it was ≈1.5-fold lower in fructose (Fig. 2H). These results provided a basis for the selection of malate (supporting a comparable yield with a faster growth rate) as an efficient carbon source over glycerol (supporting a slightly higher yield than malate but the slowest growth rate) and fructose (supporting a slower growth rate than malate and the lowest carotenoid yield).

### Analysis of metabolic efficiency of malate for isoprenoid biosynthesis

**Fig 3:**
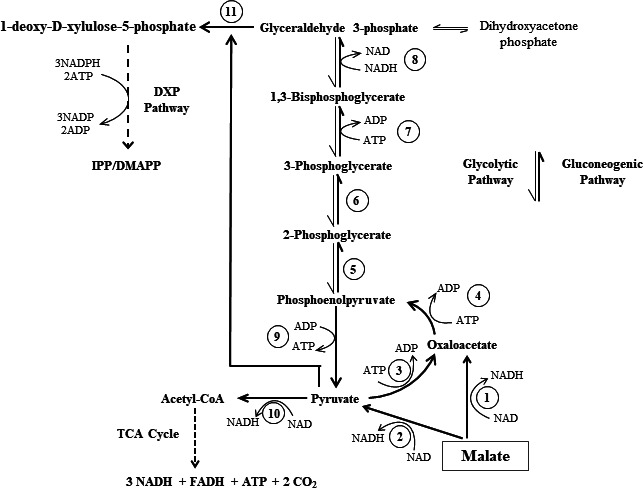
Schematic representation of the central metabolic (glycolytic, gluconeogenic, and TCA cycle) pathway reactions (adapted from reference 23) involved in the utilization of malate for DXP pathway-mediated biosynthesis of IPP and DMAPP. Thin arrows indicate the glycolytic reactions not essential for malate utilization. Encircled numbers indicate the enzymes: 1, malate dehydrogenase; 2, malic enzyme; 3, pyruvate carboxylase; 4, phosphoenolpyruvate (PEP) carboxykinase; 5, enolase; 6, phosphoglycerate mutase; 7, phosphoglycerate kinase; 8, glyceraldehyde 3-phosphate dehydrogenase; 9, pyruvate kinase; 10, pyruvate dehydrogenase; and 11, 1-deoxy-D-xylulose-5-phosphate synthase. Malate is given in a rectangular box to indicate its supply from the media. Dotted arrows indicate multistep enzyme pathways.

Although MMA has been used as a preferred medium for *A. brasilense* (12), the malate utilization efficiency of this bacterium has not been evaluated. Malate estimation revealed that the stationary phase cultures (≈6.50 OD_600 nm_) of AK04 contained only 2%–5% of the malate that was present in the fresh media, indicating >95% (>7.6 g/L of 8.0 g/L) of the malate consumption (data not shown). Next, we examined its metabolic efficiency by analyzing the central carbon metabolic pathways involved in the utilization of malate for IPP/DMAPP through the DXP pathway, which requires one molecule of glyceraldehyde 3-phosphate (G3P) and pyruvate (as substrates), and 2 ATP and 3 NADPH (as cofactors) for each IPP/DMAPP (22). Figure 3 shows that malate can enter the metabolism via two routes: (i) through malate dehydrogenase (a TCA cycle enzyme) mediated oxidation to oxaloacetate, which can be converted to G3P and pyruvate through glycolytic/gluconeogenic routes (23); and (ii) through malic enzymes-mediated decarboxylation to pyruvate (24), which can be converted to G3P through the gluconeogenic route. The malate-derived pyruvate can enter the DXP pathway as such or TCA cycle through acetyl-CoA. Stoichiometric analysis suggests the requirement of three malate molecules for the biosynthesis of each IPP/DMAPP (Fig. S2); two for the biosynthesis of one molecule of each substrate (pyruvate and G3P), while one for the production of the required cofactors (NADPH and ATP) by its complete catabolism through the TCA cycle and the electron transport chain. This suggests that 9 moles of malate is required for the biosynthesis of 1 mole of FPP (2 IPP +1 DMAPP) or sesquiterpene, since STPS-mediated FPP to sesquiterpene conversions follow an equimolar stoichiometry. Since carotenoids produced by *A. brasilense* have not yet been identified*,* the number of moles cannot be calculated from their total yield (estimated in the above result), and hence, the requirement of malate for the achieved carotenoid yield cannot be estimated.

### Validation of activities of the enzymes encoded by optimized/synthesized genes

Based on the reported efficiencies, valencene synthase (CnVS) of *C. nootketensis* (6) and α-humulene synthases of *Zingiber zerumbet* (ZSS1) (25) and *Aquilaria crassna* (AcHS2) (26) were selected to be expressed in *A. brasilense*. Since the GC content in the *A. brasilense* Sp7 genome is >67%, the selected genes were codon-optimized (Table S2), synthesized, and cloned in pET28a to construct pAK052 (pET28a-*CnVS*), pAK053 (pET28a-*ZSS1*), and pAK060 (pET28a-*AcHS2*). Proteins were expressed separately from each of the constructs in *E. coli* BL21(DE3) pLysS (Fig. S3A and S4A), and *in vitro* bioassays were performed with soluble fractions using FPP as a substrate. Hexane extract of these assays showed GC peaks corresponding to the valencene with the protein expressed from pAK052 (Fig. S3B), and to the α-humulene with the proteins expressed from pAK053 or pAK060 (Fig. S4B). Mass spectrum of the peak obtained with pAK052 matched with that of the authentic valencene standard (Fig. S3C and D), while spectra of the peaks obtained with pAK053 or pAK060 matched with that of the α-humulene standard (Fig. S4C through E). These results show that the synthesized ORFs encode active enzymes.

### Expression of sesquiterpene biosynthetic genes in *A. brasilense* strains

**Fig 4:**
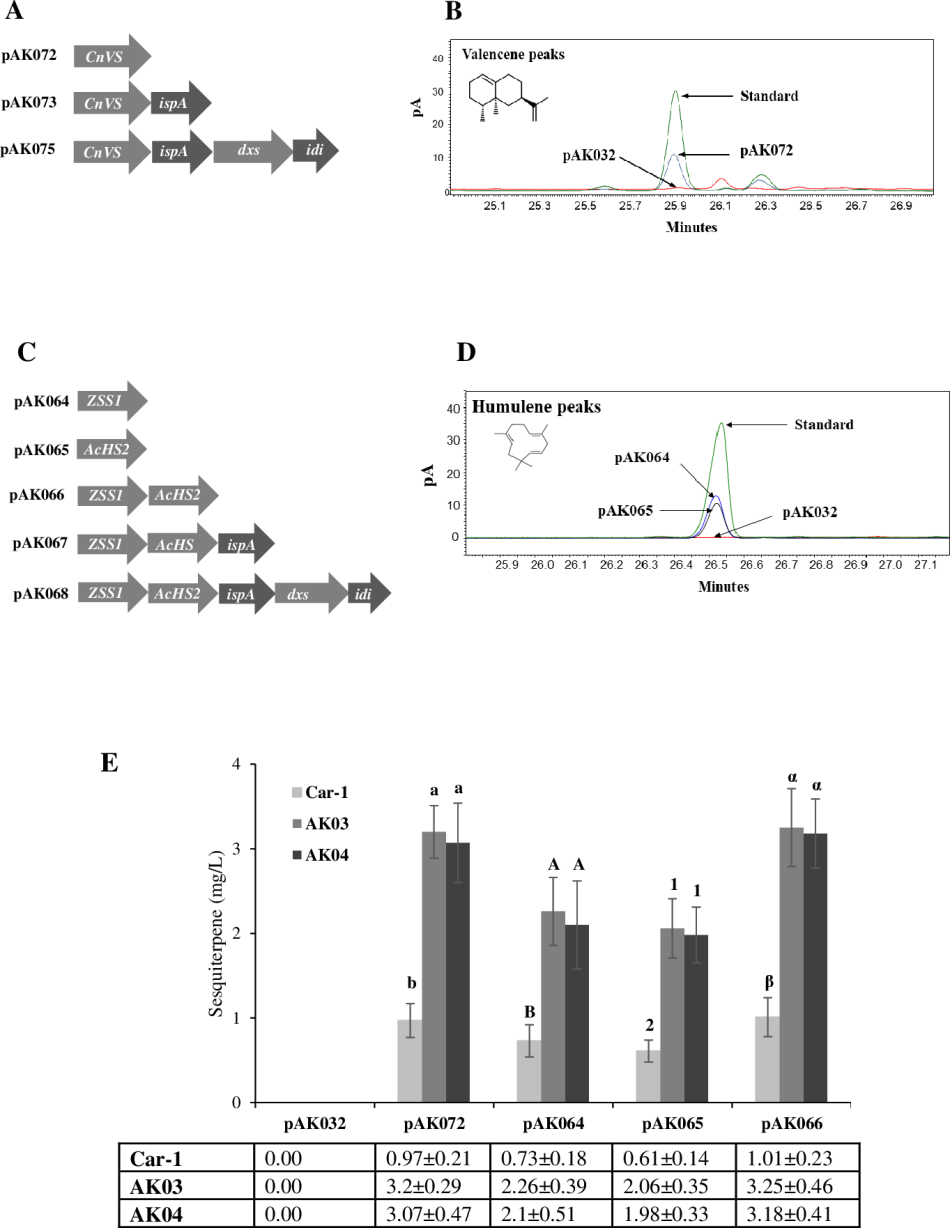
Schematic presentation of the genes present in different plasmids constructed for valencene (**A**) and α-humulene (**C**) biosynthesis; valencene synthase (*CnVS*) of *Callotropis nootketensis*, α-humulene synthase of *Zingiber zerumbet* (*ZSS1*) and *Aquilaria crassna* (*AcHS2*), farnesyl pyrophosphate synthase (*ispA*) and IPP isomerase (*idi*) of *E. coli*, and 1-deoxy-D-xylulose 5-phosphate synthase (*dxs*) of *A. brasilense*. GC chromatogram of the dodecane extracts of the AK03 cultures harboring pAK032 or pAK072 (**B**), and pAK032, pAK064, or pAK065 (**D**); chromatograms of the respective standards are included in each case. (**E**) Comparison of sesquiterpene-producing efficiencies of Car-1, AK03, and AK04 by expression of different sesquiterpene synthases. Each bar shows the mean and standard deviation of values obtained from the triplicate experiments, and *P*-values < 0.05 were considered for significant differences. Different letters show the statistically significant differences in the sesquiterpene production in Car-1, AK03, and AK04 by pAK072 (lowercase letters), pAK064 (uppercase letters), pAK065 (numbers), and pAK066 (α & β).

Since sesquiterpenes are known to inhibit microbial growth (27), and since 10% n-dodecane is used for *in situ* separation of the produced sesquiterpenes (9), we examined the tolerance level of *A. brasilense* strains to sesquiterpenes and dodecane. This reveals that the growth of neither of the strains was affected by up to 1.0 g/L of valencene/humulene and 10% n-dodecane, indicating an intrinsic sesquiterpene tolerance and biocompatibility of n-dodecane for *A. brasilense*. To examine the effect of improved carotenoid flux on sesquiterpene production, codon-optimized STPS genes were expressed in Car-1, AK03, and AK04. Analysis revealed that AK03 harboring pAK072 (pAK032-*CnVS*) produced a valencene-specific peak and a yield of 3.2 ± 0.31 mg/L (Fig. 4A and B), which was >3-fold higher than the Car-1/pAK072 (0.97 ± 0.2 mg/L) (Fig. 4E). However, AK04/pAK072, which was supposed to produce a higher yield because of its higher carotenoid productivity than the AK03, could produce a yield only equal to the AK03/pAK072. Furthermore, the AK04/pAK072 strain was not stable on MMA or LB solid media for more than 2–3 generations, and the same issue was observed when α-humulene biosynthesis was attempted in the AK04 using pAK064 (pAK032-*ZSS1*) or pAK065 (pAK032-*AcHS2*). This might be a consequence of the accumulation of toxic levels of the sesquiterpenes in AK04 due to its twofold higher carotenoid flux than AK03. AK03 harboring pAK064 or pAK065 (Fig. 4C) produced α-humulene-specific peaks (Fig. 4D) and yields of 2.26 ± 0.41 and 2.06 ± 0.35 mg/L, respectively, which w ere >3-fold higher than the yields achieved by these plasmids in Car-1 (Fig. 4E). Co-expression of the humulene synthases in AK03 using pAK066 (pAK032-*ZSS1-AcHS2*) produced a yield of 3.25 ± 0.46 mg/L, which was slightly higher than the yields achieved by their independent expressions.

**Fig 5:**
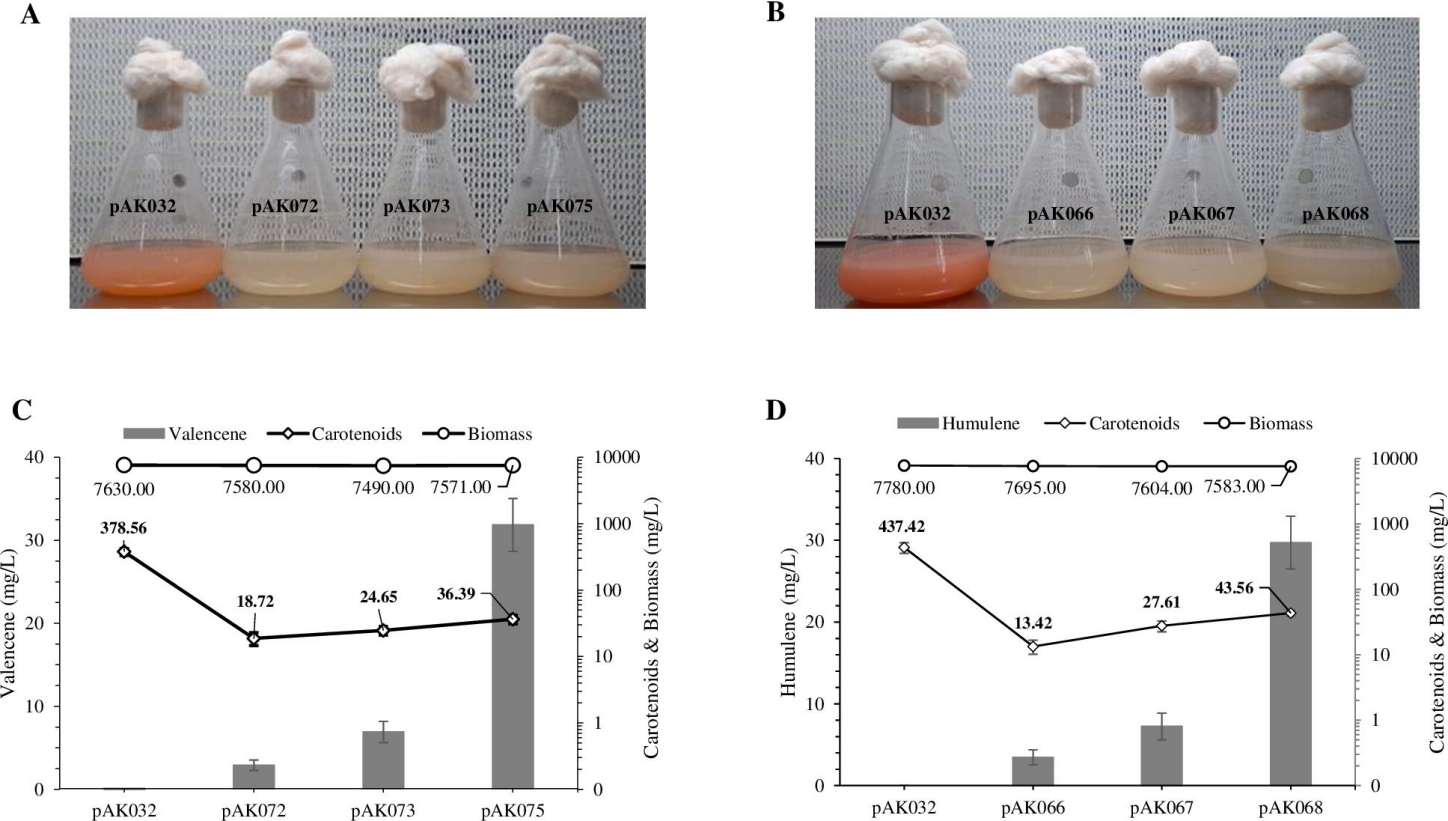
Image of culture flasks during production of valencene (**A**) and α-humulene (**B**) by AK03 harboring different expression constructs. Effect of co-expression of sesquiterpene synthases and rate-limiting enzymes (DXS, IspA, and IDI) of the DXP pathway on valencene (**C**) and α-humulene (**D**), as well as on carotenoids and biomass yields in AK03. pAK032 (empty vector), pAK072 (pAK032-*CnVS*), pAK073 (pAK032-*CnVS-ispA*), pAK075 (pAK032-*CnVS-ispA-dxs-idi*), pAK066 (pAK032-*ZSS1-AcHS2*), pAK067 (pAK032-*ZSS1-AcHS2-ispA*), and pAK068 (pAK032-*ZSS1-AcHS2-ispA-dxs-idi*). Production experiments were repeated at least three times to confirm the reproducibility. Each bar and data point shows the mean and standard deviation of values obtained from three replicates, and *P*-values < 0.05 were considered for significant difference.

Although these results indicate that improvement in the carotenoid yield improves the sesquiterpene production, the achieved sesquiterpene yields by AK03 were <1% of its carotenoid yield (400 mg/L). Since carotenoid biosynthesis goes through FPP, which is a substrate for the STPSs, and since AK03 produces >400 mg/L carotenoid, we speculated that the catalytic efficiencies of the STPSs might be a limiting factor, and if so, STPSs expression-mediated only 1% diversion of the isoprenoid flux toward the sesquiterpenes should not affect the carotenoid production significantly. However, monitoring of the fermentation cultures revealed that sesquiterpene-producing AK03 cultures stop carotenoid production almost completely (Fig. 5A and B). It indicates toward sesquiterpene-mediated downregulation of the isoprenoid/carotenoid pathway in *A. brasilense* and provides an explanation of why the sesquiterpene yields in AK03 were <1% of its carotenoid yield. Altogether, these results indicate that (i) improvement in the carotenoid flux improves the sesquiterpene yields, as the AK03 produces higher yields than the Car-1 and (ii) sesquiterpene production downregulates the isoprenoid/carotenoid pathway in *A. brasilense*. Since AK04 strains harboring STPSs expression plasmids were less stable and produced yields equal to those of the AK03, only the latter was selected for further production/engineering works.

### Engineering to increase the flux toward FPP

Sesquiterpene-mediated downregulation of the carotenoid flux prompted us to examine whether co-expression of the DXP pathway rate-limiting enzymes could improve the sesquiterpene yields. 1-deoxy-D-xylulose 5-phosphate synthase (DXS) and isopentenyl-diphosphate isomerase (IDI) have been demonstrated as major rate-limiting enzymes of the DXP pathway (2). Since *A. brasilense* IspA produces FPP as well as GGPP (13), we selected *E. coli* FPP synthase (IspA), which produces only FPP (4), to co-express with DXS and IDI to improve the FPP pool. As is obvious from Fig. 5C and D, co-expression of the IspA and STPSs using pAK073 (pAK032-*CnVS-ispA*) or pAK067 (pAK032-*zss1-AcHS2-ispA*) improves the yields ≈2-fold in comparison to the AK03 expressing only the corresponding STPSs via pAK072 or pAK066, respectively. Interestingly, pAK075 (pAK032-*CnVS-ispA-dxs-idi*) and pAK068 (pAK032-*zss1-AcHS2-ispA-dxs-idi*), which co-express each of the rate-limiting enzymes, produced 31.85±3.18 mg/L valencene and 29.72 ± 3.23 mg/L humulene, respectively, which are ≈10-fold higher than the yields achieved by expressing only the corresponding STPSs (Fig. 5C and D; Table 2). This indicates that co-expressions of the rate-limiting enzymes bypass the sesquiterpene-mediated downregulation and improve the isoprenoid flux. More importantly, it also suggests that the IspA, IDI, and DXS might be the target enzymes for sesquiterpene-mediated downregulation of the isoprenoid pathway in AK03.

**TABLE 2:** Sesquiterpene yields produced by AK03 harboring different constructs

Designation	Genotype	α-Humulene		Valencene	
		mg/L	mg/g biomass	mg/L	mg/g biomass
pAK032	Empty vector	0.0	0.0	0.0	0.0
pAK064	*ZSS1*	2.26	0.29		
pAK065	*AcHS2*	2.06	0.27		
pAK066	*ZSS1-AcHS2*	3.64	0.47		
pAK067	*ZSS1-AcHS2-ispA*	7.23	0.95		
pAK068	*ZSS1-AcHS2-ispA-dxs-idi*	29.72	3.92		
pAK110	*ZSS1-AcHS2-ispA-dxs-idi-AbtolC1*	48.71	7.13		
pAK111	*ZSS1-AcHS2-ispA-dxs-idi-AbtolC2*	49.63	7.41		
pAK072	*CnVS*			2.89	0.38
pAK073	*CnVS-ispA*			6.91	0.92
pAK075	*CnVS-ispA-dxs-idi*			31.85	4.21
pAK108	*CnVS-ispA-dxs-idi-AbtolC1*			53.61	7.99
pAK109	*CnVS-ispA-dxs-idi-AbtolC2*			51.04	7.42

It was also noted that although the co-expression improves the sesquiterpene yields, the carotenoid level in AK03 expressing STPSs alone or with the rate-limiting enzymes is almost the same (Fig. 5A and B). The co-expression-mediated enhancement in the sesquiterpene yields indicates an improvement in the isoprenoid flux, which is also supposed to improve the carotenoid yields. However, the lack of improvement in the carotenoid level of AK03 even after the co-expression suggests that the sesquiterpenes also downregulate the carotenoid biosynthetic enzyme(s), which could not be relieved by expression of the DXP pathway rate-limiting enzymes. Although the co-expression-mediated achieved yields (31.85 ± 3.18 mg/L or 29.72 ± 3.23 mg/L) are still <10% of the total carotenoid yield in AK03 (400 mg/L), ≈10-fold improvement in the yields suggests that the sesquiterpene production in AK03 can be enhanced further by increasing the FPP pool.

### Engineering to inhibit FPP utilization for endogenous carotenoid biosynthesis

**Fig 6:**
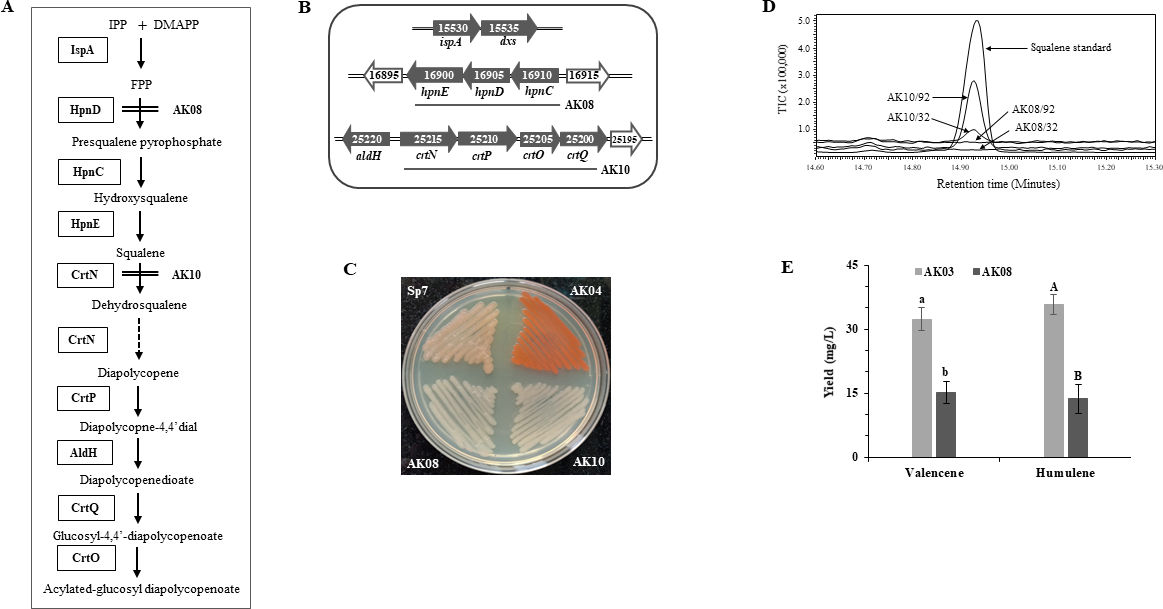
(**A**) Squalene-mediated carotenoid biosynthesis pathway in *Azospirillum brasilense* proposed by an earlier report (28) and validated by the present study. Points of blockage in the pathway due to the deletion of *hpnCDE* and *crtNPOQ* are depicted by horizontal double lines. (**B**) Schematic representation of *Azospirillum brasilense* Sp7 chromosomal regions encoding putative homologs of the reported enzymes (names given below the corresponding arrows) involved in C30 carotenoid biosynthesis. White arrows represent the flanking ORFs not involved in the carotenoid pathway; superoxide dismutase (16895), hypothetical protein (16915), and pseudogene (25195). Numbers in the horizontal arrows indicate the last five digits of their locus tags, such as 25215 indicates AMK58_25215. Horizontal single lines depict the chromosomal region deleted to construct *ΔhpnCDE* (AK08) and *ΔcrtNPOQ* (AK10) mutants. (**C**) Luria agar plate showing colonies of Sp7 (parent), AK04 (*ΔchrR1*), AK08, and AK10. (**D**) GC-MS chromatogram of the organic extracts prepared from AK08 and AK10 cells harboring pAK032 (empty vector) or pAK092 (pAK032-*rpoE1*) extracts; TIC represents total ion chromatogram. (**E**) Comparison of sesquiterpene yields in AK03 and AK08 strains using pAK075 (for valencene) and pAK068 (for humulene) constructs. Each bar shows the mean and standard deviation of values obtained from the three replicates, and *P*-values < 0.05 were considered for significant difference. Different letters show the statistically significant differences in valencene (lowercase letters) and humulene (uppercase letters) production by AK03 and AK08.

The carotenoid pathway can be truncated to improve the FPP pool if carotenoids are dispensable to bacteria. Although downregulation of the carotenoid pathway in *A. brasilense* Sp7 has been reported earlier (12), complete dispensability of the carotenoids has not yet been examined. Recently, a squalene-mediated carotenoid pathway was reported in *A. brasilense* Cd (28); however, squalene has not yet been detected as an intermediate metabolite. Genome analysis revealed that Sp7 encodes homologs of the enzymes involved in the squalene-mediated carotenoid pathway (Fig. 6A) (29), and squalene biosynthetic genes (*hpnCDE*) are clustered at one locus while downstream genes (*crtNPOQ* and *aldH*) of the pathway are clustered at a different locus (Fig. 6B). We attempted to delete these loci in AK04, but mutants could not be isolated even after >10 attempts. We speculated that deletion-mediated toxic-level accumulation of the pathway intermediates (FPP and squalene) might be a reason, since AK04 carries a high-flux carotenoid pathway. This speculation was supported by the isolation of *ΔhpnCDE* (AK08) and *ΔcrtNPOQ* (AK10) mutants from Sp7, which produces only a basal level of the carotenoids. Figure 6C clearly shows that carotenoid production in AK08 and AK10 strains was abolished, as they lack the light pink color present in Sp7; extraction and estimation analysis validated the abolition of carotenoids from these strains (Fig. S5B). GC-MS analyses revealed that the AK10 accumulated squalene, and as expected, the accumulation was increased upon pAK092-borne RpoE1 expression, which is known to upregulate the carotenoid pathway (Fig. 6D; Fig. S5A). Squalene-specific peaks were not detected in the samples prepared from AK08 harboring pAK032 or pAK092. These results not only validate the presence of squalene-mediated and completely dispensable carotenoid pathway in *A. brasilense* Sp7 but also establish the involvement of *hpnCDE* and *crtNPOQ* clusters in the carotenoid biosynthesis.

Since HpnCDE mediates the FPP utilization for carotenoid biosynthesis, the AK08 was selected as an FPP-providing platform strain for sesquiterpene production. Since the AK08 was constructed from Sp7, constructs were made to co-express the RpoE1 with the valencene biosynthetic operon in AK08. For this, the *rpoE1* was inserted into pAK072 and pAK075 to construct pAK105 (pAK032-*CnVS-rpoE1*) and pAK107 (pAK032-*CnVS-ispA-dxs-idi-rpoE1*), respectively. Surprisingly, only pAK075 could produce valencene in AK08 (Fig. S4C). These results suggest that (i) the AK08 does not accumulate FPP, as pAK072 (expressing only CnVS) could not produce valencene and (ii) it does not accumulate the FPP even during RpoE1 expression-mediated upregulation of the carotenoid pathway, since pAK105 and pAK107 (co-expressing RpoE1) either could not produce or produced a very small amount, respectively. However, the pAK075-mediated valencene production suggests that *dxs-idi* co-expression makes the FPP pool available for valencene synthesis. Given the pAK075-mediated valencene production in AK08, the sesquiterpene-producing potential of AK08 was compared with that of AK03 by expressing pAK075 and pAK068 in both strains. Figure 6E shows that the valencene or humulene yield in AK08 was half of that achieved in AK03.

The above results prompted us to examine the effect of *hpnCDE* deletion and RpoE1 expression on the FPP level in *A. brasilense* Sp7 by measuring its level (as farnesol) in the parent and AK08 cells harboring empty (pAK032) or the RpoE1 expressing (pAK092) plasmids. Intriguingly, the GC-MS analysis could not detect farnesol in the extracts prepared from either of the strains harboring either of the plasmids (Fig. S5). Considering that FPP is an intermediate of the carotenoid pathway, the lack of FPP in Sp7/pAK092, which produces carotenoids, was surprising. To validate this, FPP detection was performed in AK03 and AK04, which produces carotenoids constitutively. However, FPP could not be detected in either of the strains (Fig. S5). Since the extracted samples were dephosphorylated to convert the FPP into farnesol for GC-MS detection, an authentic FPP standard was dephosphorylated, and the produced farnesol was positively detected to rule out any dephosphorylation-related issues. The lack of FPP in AK08/pAK092 might be a consequence of a tight feedback inhibition of the isoprenoid pathway in response to the toxic level of FPP accumulation due to RpoE1-mediated upregulation of the pathway and lack of the FPP-utilizing enzymes (HpnCDE). However, the lack of FPP in the carotenoid-producing strains (Sp7/pAK092, AK03, and AK04) suggests that this bacterium does not accumulate FPP even in the high-level carotenoid-producing strains.

### Engineering to improve extracellular transport

**Fig 7:**
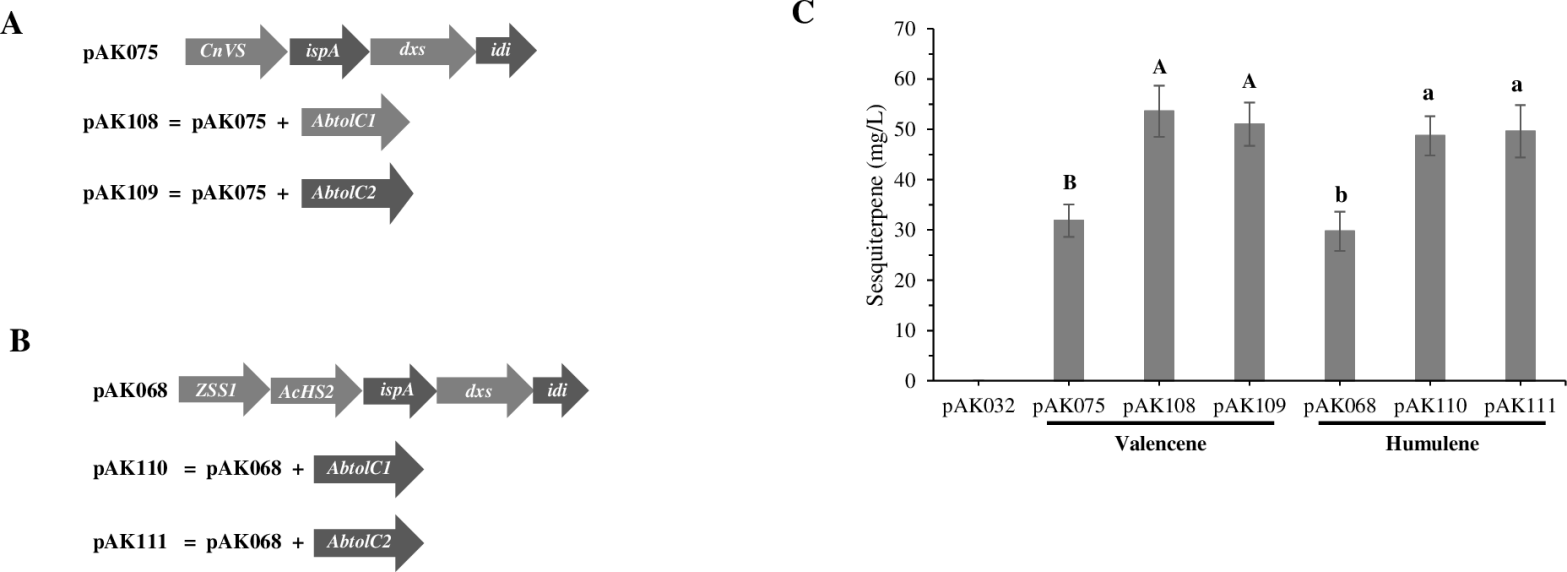
Schematic presentation of the transporter-encoding genes inserted in pAK075 (**A**) and pAK068 (**B**) to construct different plasmids for their co-expression with the sesquiterpene synthases; *AbtolC1* and *AbtolC2*, encoding homologs of *E. coli* TolC (an outer membrane protein). (**C**) Effect of co-expression on the sesquiterpene production by AK03. Each bar shows the mean and standard deviation of values obtained from the triplicate experiments, and *P*-values < 0.05 were considered for significant differences. Different letters show the statistically significant differences in valencene (uppercase letters) and humulene (lowercase letters).

Sesquiterpene-mediated downregulation of the carotenoid flux prompted us to examine whether extracellular transport engineering could improve the yields. Out of several studied pumps, TolC (an outer membrane component of efflux pumps in *E. coli*) has been reported to make a significant improvement in sesquiterpene yields (30, 31). Since heterologous expression of many efflux pumps is less effective than the native orthologs (32), we performed genome analysis to identify TolC orthologs in *A. brasilense*. This revealed that TolC shares 24% and 22% identity with two putative outer membrane proteins (OMP) encoded by AMK58_07740 and AMK58_14050 (hereafter designated *AbtolC1* and *AbtolC2*, respectively) loci in the *A. brasilense* genome, respectively. *AbtolC1* and *AbtolC2* were inserted individually into pAK075 and pAK068 to construct plasmids for their co-expression with the valencene and humulene biosynthetic operons, respectively (Fig. 7A and B). Production experiment revealed that their co-expression improved the sesquiterpenes yield >1.5-fold in comparison to the constructs (pAK075 or pAK068) without efflux proteins (Fig. 7C, Table 2). *AbtolC1* and *AbtolC2* expression-mediated almost equal improvement is in agreement with their similar functional nature, since both of them are supposed to work as OMPs as TolC does in *E. coli* (32). To validate their expression, translationally fused *AbtolC1-gfp* and *AbtolC2-gfp* ORFs were inserted into pAK075 to construct pAK115 and pAK116, respectively (Fig. S7A). Since the fused *gfp* ORFs, which are without independent RBS and start codon, could only be expressed if *AbtolC1* or *AbtolC2* are translated, GFP expression in AK03 harboring pAK115 or pAK116 validates *AbtolC1* and *AbtolC2* expression, respectively (Fig. S7B and C). These results highlight the importance of extracellular transport engineering to improve sesquiterpene production in *A. brasilense*.

## DISCUSSION

The ever-increasing commercial applications have increased the demand for several sesquiterpenes, and limitations associated with extraction from plants or chemical synthesis have shifted the interest toward their heterologous production using engineered microbes. Although several microbes have been engineered, most of them lack the industrial potential yield (9). The major issues are as follows: (i) lack of sufficient starting substrate (FPP); (ii) lack of efficient/robust STPSs; (iii) lack of efficient/optimized STPS expression; and (iv) intracellular accumulation of sesquiterpenes to toxic levels due to their insufficient extracellular transport (31). Although different approaches are being used to address these issues, a lack of sufficient FPP pool is still a major issue, as most of the microbial hosts (including the most commonly used *Saccharomyces cerevisiae* and *E. coli*) require tedious engineering for this. To address this, sesquiterpene-producing potential of microbes carrying endogenous high-flux isoprenoid pathways is being explored (2, 6, 33). However, a lack of sufficient physio-genetic details as well as developed genetic tools limits their exploration. With these considerations, we initiated this study based on a hypothesis that a bacterium carrying an intrinsic high-flux carotenoid pathway might require less engineering for the FPP pool and provide an opportunity to deal with other issues, such as engineering for extracellular transport. For this, we have constructed AK03 (*chrR1*::Km^R^) and AK04 (*ΔchrR1*) as high-level carotenoid-producing strains from *A. brasilense* Sp7 by utilizing FLP/FRT recombination system for the first time in this bacterium and explored the sesquiterpene production using α-humulene and (+)-valencene as two model compounds.

Although carotenoid and terpene production in *A. brasilense* Sp7 has been shown using Car-1 (*chrR1*::mTn*5*) strain (12, 13), carotenoid-producing potential, which directly reflects the strength of isoprenoid flux, of this bacterium has not yet been examined. Our observation that AK03/AK04 produce higher biomass and carotenoids than the Car-1 clearly indicates an mTn*5*-mediated polar effect in Car-1, and rules out the possibility of any negative effect of *chrR1* deletion or constitutive carotenoid production on the growth/biomass. The mTn5-mediated polar effect might be a consequence of its insertion into *chrR1*, which is transcriptionally and translationally coupled with the upstream *rpoE1* encoding a positive regulator of the carotenoid biosynthesis (12). This hypothesis is supported by our observation that AK03, which has a Km^R^ cassette in the *chrR1* ORF in a transcriptionally opposite direction, produces ≈2-fold less carotenoid yield than AK04, which is an unmarked *chrR1* mutant. Interestingly, media optimization data showing >0.8 g/L carotenoid yield in AK04 suggests that *A. brasilense* Sp7 has evolved to carry a high endogenous carotenoid flux without compromising its growth rate and biomass yield.

Rich media supplemented with additional carbon sources, such as LB with glycerol for *E. coli* (4, 34) and YPD with glucose/galactose for *S. cerevisiae* (9, 35), have extensively been used for microbial fermentations; however, the use of a single carbon source-based minimal media is rare (33). Since *A. brasilense* does not utilize glucose (21), an efficient carbon source is required to work with this bacterium. Our data establish malate as an efficient carbon source for *A. brasilense* by showing better efficiency of MMA over rich or glycerol/fructose-based minimal media, and consumption of >95% (>7.6 g/L out of 8.0 g/L) malate present in the modified-MMA. Based on the stoichiometric analysis, we have estimated that 9 moles of malate is required for the biosynthesis of each mole of FPP or sesquiterpene. However, due to a lack of information about the carotenoids produced by *A. brasilense,* the requirement of malate for the achieved carotenoid yield cannot be estimated. Although an earlier report (28) has suggested and our data validate the presence of squalene-mediated carotenoid pathway in this bacterium, the intermediates/terminal carotenoids, which go through different types (oxidation, glycosylation, acylation, etc.) and degrees of modifications (29, 36), are not known.

Extensive engineering, which includes expression of the rate-limiting enzymes, global metabolic rewiring, coupling cell growth and biochemical pathway, etc., has developed a *S. cerevisiae* strain producing >16 g/L valencene (35). However, expression of CnVS in the yeast without the additional engineering could produce only 1.36 and 11.6 mg/L valencene in WAT11 (6) and W303 (9) strains, respectively. Furthermore, CnVS expression in the engineered carotenoid-producing bacteria has achieved 2.41, 18, and 352 mg/L valencene in *C. glutamicum* (27), *R. capsulatus* (2), and *Rhodobacter sphaeroides* (6), respectively. However, the expression without additional engineering could produce an appreciable amount (57 mg/L) only in *R. sphaeroides* (6); it produced <1 mg/L in *R. capsulatus* and an undetectable amount in *C. glutamicum*. α-humulene production has been explored in *E. coli* (37), *Methylobacterium extorquens* (33), and *S. cerevisiae* (7), and although ZSS1 with additional engineering produced >1.0 g/L in the first two and 28 mg/L in the third microbe, only ZSS1 expression could produce <5.0 mg α-humulene in these microbes. Our data show that the expression of CnVS, ZSS1, or AcHS2 in AK03 produces the corresponding sesquiterpenes between 2 and 3 mg/L, which are either comparable or higher than the reported yields without additional engineering. However, these are less than the yield achieved by expressing amorphadiene synthase (ADS) in Car-1 in our earlier study (13), which seems mainly due to efficient activity of the ADS, as heterologous amorphadiene production has been achieved several folds higher than the humulene or valencene (38).

Our observation that the achieved sesquiterpene yields in AK03 are <1% of its total carotenoid yield indicates that the STPSs expression diverts a very small portion of the isoprenoid flux toward the sesquiterpenes. However, the lack of carotenoid production by the sesquiterpene-producing cultures provides an explanation for the low yield by suggesting that the heterologous sesquiterpenes downregulate the isoprenoid/carotenoid pathway in AK03. Interestingly, 10-fold improvement in the sesquiterpene yields by co-expression of the rate-limiting enzymes with either of the STPSs not only highlights the rate-limiting nature of DXS, IDI, and IspA in *A. brasilense* but also suggests that these enzymes may be the potential target for sesquiterpene-mediated downregulation of the isoprenoid flux in this bacterium. However, sesquiterpene-mediated inhibition of other enzymes of the DXP pathway cannot be excluded. Our data also show that although AK04 produces twofold higher carotenoid than the AK03, sesquiterpene yields by AK04 were not more than AK03, and the presence of STPSs expressing-plasmids renders AK04 unstable on the solid media, which might be a consequence of the accumulation of toxic levels of sesquiterpenes (that cannot be diffused away from the cells on the solid media) in the AK04 cells due to its higher carotenoid flux than the AK03.

The dispensability of carotenoids in microbes provides the opportunity to truncate the carotenoid pathway downstream of desired intermediates (such as FPP, GGPP, phytoene, etc.) to improve their availability for heterologous enzymes. Implementation of this strategy improved valencene and α-humulene yield in *C. glutamicum* (27) and *M. extorquens* (33), respectively. Our data showing *hpnCDE* or *crtNPOQ* deletion-mediated abolition of carotenoids, and squalene accumulation upon deletion of the latter locus, provides evidence for the presence of a squalene-mediated and completely dispensable carotenoid pathway in *A. brasilense* Sp7. However, *hpnCDE* deletion, which truncates the pathway downstream to FPP, could not improve the sesquiterpene yield, which might be a consequence of tight feedback inhibition of the DXP pathway in response to FPP accumulation. This hypothesis was supported by our two observations: (i) although the carotenoid pathway could be truncated in the parent strain, which produces only a basal level of carotenoids, it could not be truncated in AK04, which produces a high level of carotenoids; and (ii) FPP could not be detected in the extracts prepared from the strains carrying either a truncated or ongoing carotenoid pathway.

Recent reports have demonstrated that efflux engineering improves heterologous sesquiterpene production, and TolC (an outer membrane component of efflux pumps in *E. coli*) is intimately involved in sesquiterpene transport (30). Sesquiterpene-mediated downregulation of the carotenoid pathway in AK03 also emphasizes the importance of extracellular transport of the sesquiterpenes. Our data showing AbTolC1 or AbTolC2 co-expression-mediated >1.5-fold improvement in the valencene and humulene yields validates the importance of efflux engineering. Since AbTolC1 or AbTolC2 (encoded by *A. brasilense*) is a potential ortholog of TolC, and since it is supposed to work only as an outer membrane component of different efflux pumps (as TolC in *E. coli*), extracellular transport of sesquiterpenes can be improved further by the identification of other components (inner membrane and membrane fusion proteins) of the sesquiterpene-specific efflux pumps and their co-expression with AbTolC1 and/or AbTolC2.

In conclusion, our study provides evidence that *A. brasilense* Sp7 carries an endogenous, high-flux, squalene-mediated, and dispensable carotenoid pathway, and it has evolved to produce a high carotenoid yield without compromising its growth parameters. However, heterologous sesquiterpene-mediated downregulation of the isoprenoid pathway limits the efficient diversion of its high isoprenoid flux toward the sesquiterpenes. Outcomes of this study suggest that the identification of sesquiterpene-specific efflux pumps and isoprenoid pathway genes/enzymes downregulated by the sesquiterpenes, and their co-expression with STPSs can resolve the issue and improve sesquiterpene production. For the first time (to our knowledge), this work provides insight into heterologous sesquiterpene-mediated downregulation of the bacterial isoprenoid pathway and highlights the importance of this issue for metabolic engineering. However, how sesquiterpenes downregulate the isoprenoid pathway is a matter of further research.

## SUPPLEMENTAL MATERIAL

Supplemental material aem.00433-26-s0001.pdf Tables S1 and S2; Fig. S1 to S7.

ASM does not own the copyrights to Supplemental Material that may be linked to, or accessed through, an article. The authors have granted ASM a non-exclusive, world-wide license to publish the Supplemental Material files. Please contact the corresponding author directly for reuse.
