# Supplementary material for: Pathway and efflux engineering to utilize endogenous high isoprenoid flux in Azospirillum brasilense Sp7 for sesquiterpene production
Source: Appl Environ Microbiol. 2026 May 21;92(6):e00433-26. doi: 10.1128/aem.00433-26 (PMC13274349; doi:10.1128/aem.00433-26)
Supplement: Supplemental material — Tables S1 and S2; Fig. S1 to S7. [file aem.00433-26-s0001.pdf]

Suppl. Table 1. Primers used in this study (restriction sites are underlined)

Primers	Sequence (5’ to 3’)
chrR1:AF:EcoRI	GGAATTCGGCTTGGCAAGCGTGGTCATCAC
chrR1:AR:BglII	GAAGATCTGGCATAGTCGATCAGCAGGGTGTC
chrR1:BF:BglII	GAAGATCTCTCAACCCGTTTGTGAAGTTCTGAG
chrR1:BR:PstI	AACTGCAGGGTCTCGGTGTCGGTCGGCAGATGC
Km:F:BamHI	CGGGATCCGATTGTGTAGGCTGGAGCTGCTTC
Km:R:BamHI	CGGGATCCGTCCACATGAATATCCTCCTTAG
flp:F:PstI	AACTGCAGATGCCACAATTTGATATATTATG
flp:R:KpnI	GGGGTACCGTACTTATATGCGTCTATTTATG
rpoE1:NdeI	GGAATTCCATATGCAGGATCTTGGTTCCCCAG
rpoE1:R:PstI	AACTGCAGGGTCAGCGGGAGTCCCTCATG
chrR1:F:Nde1	GGAATTCCATATGACCGTGCCCACCCACCATC
chrR1:R:PstI	AACTGCAGGATCCGGCTCAGAACTTCACAAAC
rpoE1:F:XhoI	CCGCTCGAGCAGAGACGGCGCCCCCGAAGACGTG
rpoE1:F:NheI	CTAGCTAGCCAGAGACGGCGCCCCCGAAGACGTG
rpoE1:R:KpnI	CGGGGTACCGGCACGGTCAGCGGGAGTCCCTCATG
CnVS:F:NdeI	GGAATTCCATATGGCCGAGATGTTCAACGGCAACTC
CnVS:R:PstI	AACTGCAGTCACGGGATGATCGGCTCGACGAAG
zss1:F:NdeI	GGAATTCCATATGGAGCGCCAGTCGATGGCCCTGGTG
zss1:R:PstI	AACTGCAGTCAGATCAGGAAGCTCTCGACGAAGATC
AcHS2:F:NdeI	GGAATTCCATATGAGCCCGGCCCAGGCCCCGCAG
AcHS2:R:KpnI	CGGGGTACCGCAGCGCTCTAGATCAGATGGTGAACGGGTGGAC
AcHS2:F:PstI	AACTGCAGCGTGCCCGAAGACGTGGATGCAGGATATGAGCCCGGCCCAGGCCCCGCAG
ispA:F:PstI	AACTGCAGCATAAACAGTAATACAAGGGGTGTATGAGCCATATTCAAATGGACTTCCCGCAGCAGCTGGAG
ispA:R:XhoI	CCGCTCGAGTCACTTGTTGCGCTGGATGATGTAGTC
Dxs:F:XhoI	CCGCTCGAGCCTGAACGGAGGACGTTTCCAACGTGAC
Dxs:R:KpnI	CGGGGTACCGCAGCGCTCTAGATTTGGAGTCAGG
Idi:F:NheI	CTAGCTAGCGTCAATCATCATTGGAGTCGACCTTGGCGGTCGTGATGCAAACGGAACACGTC
Idi:R:KpnI	CGGGGTACCGCAGCGCTCTAGAGTCGGGGTTTTTTTATTTAAGCTG
hpnC:USF:Pst1	AACTGCAGGGAAGGTTCTGATGGTGTCCATGTC
hpnC:USR:KpnI	CGGGGTACCGACGAACCGGTAGAAGGCCATGACATG
hpnE:DSF:KpnI	CGGGGTACCGATGGGGCTTCCGGCAACACTCGAAG
hpnE:DSR:XbaI	TGCTCTAGAAGATGGCGGACATCACGCCGATCATC
Km:F:KpnI	CGGGGTACCGATTGTGTAGGCTGGAGCTGCTTC
Km:R:KpnI	CGGGGTACCGTCCACATGAATATCCTCCTTAG
crtN:USF:EcoRI	GGAATTCTCACGTCACAGTGGTCTCCGGGTCAGTC
crtN:USR:BglII	GAAGATCTTCAGAGCATCGCGGATGCCAATCCAC
crtQ:DSF:BglII	GAAGATCTCCTGATCCTGCTGGCGATCCAATG
crtQ:DSR:PstI	AACTGCAGGTGTGGCTGATCATCCTCTCAGAC
AbtolC1:F:XbaI	TGCTCTAGAGAATCGACCCGAACATGAAGGTC
AbtolC1:R:KpnI	CGGGGTACCAGGGCGCTCAAGTGCGTTCACTC
AbtolC2:F:XbaI	CTATCTAGACTGACCGGAGCGCCACAATCTCTAG
AbtolC2:R:KpnI	CGGGGTACCTCAGCCGCTCATCGCGGGTCTCCCGTTC
AbtolC1(gfp):R	CTCCTCGCCCTTGCTCACCTCCGGCACGCCGGTTCCGATC
gfp(abtolC1):F	GATCGGAACCGGCGTGCCGGAGGTGAGCAAGGGCGAGGAG
AbtolC2(gfp):R	CTCCTCGCCCTTGCTCACTCGCGGGTCTCCCGTTC
gfp(abtolC2):F	GAACGGGAGACCCGCGAGTGAGCAAGGGCGAGGAG
gfp:R:KpnI	CGGGGTACCTTACTTGTACAGCTCGTCCA

Suppl. Table 2. Nucleotide sequences of codon-optimized open reading frames of the sesquiterpene synthases used in this study.

>CnVS

ATGGCCGAGATGTTCAACGGCAACTCCTCGAACGACGGCAGCTCCTGTATGCCGGTGAAGGACGCCCTGCGTCGCACCGGCAACCACC
ACCCGAACCTGTGGACCGACGACTTCATCCAGTCCCTGAACTCGCCGTACAGCGACTCCTCCTACCACAAGCACCGCGAGATCCTGAT
CGACGAGATCCGCGACATGTTCTCCAACGGCGAGGGCGACGAGTTCGGCGTGCTGGAGAACATCTGGTTCGTGGACGTGGTCCAGCGC
CTGGGCATCGACCGCCACTTCCAGGAAGAGATCAAGACCGCCCTGGACTACATCTACAAGTTCTGGAACCACGACAGCATCTTCGGCG
ACCTGAACATGGTGGCCCTGGGCTTCCGCATCCTGCGCCTGAACCGCTACGTGGCCTCCTCGGACGTCTTCAAGAAGTTCAAGGGCGA
GGAAGGCCAGTTCTCCGGCTTCGAGTCCTCCGACCAGGACGCCAAGCTGGAGATGATGCTGAACCTGTACAAGGCCTCGGAGCTGGAC
TTCCCGGACGAGGACATCCTGAAGGAAGCCCGCGCCTTCGCCTCGATGTACCTGAAGCACGTCATCAAGGAGTACGGCGACATCCAGG
AGAGCAAGAACCCGCTGCTGATGGAGATCGAGTACACCTTCAAGTACCCGTGGCGCTGCCGCCTGCCGCGCCTGGAGGCCTGGAACTT
CATCCACATCATGCGCCAGCAGGACTGCAACATCTCCCTGGCCAACAACCTGTACAAGATCCCGAAGATCTACATGAAGAAGATCCTG
GAGCTGGCCATCCTGGACTTCAACATCCTCCAGTCGCAGCACCAGCACGAGATGAAGCTGATCAGCACCTGGTGGAAGAACTCCTCCG
CCATCCAGCTGGACTTCTTCCGCCACCGCCACATCGAGTCCTACTTCTGGTGGGCCTCGCCGCTGTTCGAGCCGGAGTTCTCCACCTG
CCGCATCAACTGCACCAAGCTGTCCACCAAGATGTTCCTGCTGGACGACATCTACGACACCTACGGCACCGTCGAGGAGCTGAAGCCG
TTCACCACCACCCTGACCCGCTGGGACGTGTCCACCGTGGACAACCACCCGGACTACATGAAGATCGCCTTCAACTTCAGCTACGAGA
TCTACAAGGAGATCGCCTCCGAGGCCGAGCGCAAGCACGGCCCGTTCGTGTACAAGTACCTGCAATCCTGCTGGAAGTCGTACATCGA
GGCCTACATGCAGGAAGCCGAGTGGATCGCCAGCAACCACATCCCCGGCTTCGACGAGTACCTGATGAACGGCGTCAAGTCCTCGGGG
ATGCGCATCCTGATGATCCACGCCCTGATCCTGATGGACACCCCGCTGTCCGACGAGATCCTGGAGCAGCTGGACATCCCGTCCTCCA
AGTCGCAGGCCCTGCTGTCCCTGATCACCCGCCTGGTGGACGACGTCAAGGACTTCGAGGACGAGCAGGCCCACGGCGAGATGGCCTC
CTCCATCGAGTGCTACATGAAGGACAACCACGGCAGCACCCGCGAGGACGCCCTGAACTACCTGAAGATCCGCATCGAGTCCTGCGTG
CAGGAGCTGAACAAGGAGCTGCTGGAGCCGTCGAACATGCACGGCAGCTTCCGCAACCTGTACCTGAACGTGGGGATGCGCGTCATCT
TCTTCATGCTGAACGACGGCGACCTGTTCACCCACAGCAACCGCAAGGAGATCCAGGACGCCATCACCAAGTTCTTCGTCGAGCCGAT
CATCCCGTGA

>ZSS1

ATGGAGCGCCAGTCGATGGCCCTGGTGGGCGACAAGGAAGAGATCATCCGCAAGTCGTTCGAGTACCACCCGACCGTCTGGGGCGACT
ACTTCATCCGCAACTACTCGTGCCTGCCGCTGGAGAAGGAGTGCATGATCAAGCGCGTGGAGGAGCTGAAGGACCGCGTCCGCAACCT
GTTCGAGGAGACCCACGACGTGCTCCAGATCATGATCCTGGTGGACAGCATCCAGCTGCTGGGCCTGGACTACCACTTCGAGAAGGAG
ATCACCGCCGCCCTGCGCCTGATCTACGAGGCCGACGTGGAGAACTACGGCCTGTACGAAGTCAGCCTGCGCTTCCGCCTGCTGCGCC
AGCACGGCTACAACCTGTCCCCGGACGTGTTCAACAAGTTCAAGGACGACAAGGGCCGCTTCCTGCCGACCCTGAACGGCGACGCCAA
GGGCCTGCTGAACCTGTACAACGCCGCCTACCTGGGCACCCACGAGGAGACCATCCTGGACGAGGCCATCAGCTTCACCAAGTGCCAG
CTGGAGTCCCTGCTGGGCGAGCTGGAGCAGCCGCTGGCCATCGAGGTCTCCCTGTTCCTGGAGACCCCGCTGTACCGTCGCACCCGCC
GCCTGCTGGTGCGCAAGTACATCCCGATCTACCAGGAGAAGGTCATGCGCAACGACACCATCCTGGAGCTGGCCAAGCTGGACTTCAA
CCTGCTCCAGTCCCTGCACCAGGAAGAGGTGAAGAAGATCACCATCTGGTGGAACGACCTGGCCCTGACCAAGTCGCTGAAGTTCGCC
CGCGACCGCGTGGTCGAGTGCTACTACTGGATCGTGGCCGTCTACTTCGAGCCGCAGTACTCCCGCGCCCGCGTCATCACCTCGAAGG
CCATCTCGCTGATGAGCATCATGGACGACATCTACGACAACTACTCGACCCTGGAGGAGTCCCGCCTGCTGACCGAGGCCATCGAGCG
CTGGGAGCCGCAGGCCGTGGACTGCGTCCCGGAGTACCTGAAGGACTTCTACCTGAAGCTGCTGAAGACCTACAAGGACTTCGAGGAC
GAGCTGGAGCCGAACGAGAAGTACCGCATCCCGTACCTGCAAGAGGAGATCAAGGTGCTGTCGCGCGCCTACTTCCAGGAAGCCAAGT
GGGGCGTGGAGCGCTACGTGCCGGCCCTGGAGGAGCACCTGCTGGTCTCGCTGATCACCGCCGGCTACTTCGCCGTGGCCTGCGCCAG
CTACGTCGGCCTGGGCGAGGACGCCACCAAGGAGACCTTCGAGTGGGTGGCCTCCTCGCCGAAGATCCTGAAGAGCTGCTCCATCCAC
TGCCGCCTGATGGACGACATCACCAGCCACCAGCGCGAGCAGGAGCGCGACCACTTCGCCTCCACCGTGGAGTCGTACATGAAGGAGC
ACGGCACCTCGGCCAAGGTGGCCTGCGAGAAGCTCCAGGTGATGGTCGAGCAGAAGTGGAAGGACCTGAACGAGGAGTGCCTGCGCCC
GACCCAGGTGGCCCGCCCGCTGATCGAGATCATCCTGAACCTGTCCCGCGCGATGGAGGACATCTACAAGCACAAGGACACCTACACC
AACAGCAACACCCGCATGAAGGACAACGTGTCGCTGATCTTCGTCGAGAGCTTCCTGATCTGA

>AcHS2

ATGAGCCCGGCCCAGGCCCCGCAGGTGTCCGCCCCGACCCAGAAGGCCGCCGACGAGGAGGCCAACCGCCGCTCGGCCGGCTACCACC
CGAGCTTCTGGGGCGAGTTCTTCCTGACCCACTCCTCGGGCTACACCAAGAGCGACACCAAGATCCAGCAGAAGCACGAGGAGCTGAA
GCAGCAGGTGCGCGGCATGATCCTGGACGCCGCCGCCGACACCAGCCAGAAGCTGGAGCTGATCGACGCCGCCCTGCGCCTGGGCGTC
GGCTACCACTTCGAGGCCGAGATCCAGTCCCAGCTCCAGAAGATCCACGGCCAGGGCTCCTTCCACTCGGACCTGTACACCGCCTGCA
TCTGGTTCCGCGTCCTGCGCGGCCAGGGCTTCACCGTGTCCGCCGACGTCTTCAACATCATGAAGAACAAGGACGGCGGCTTCGAGGC
CCGCGACGCCCGCACCCTGCTGTGCCTGTACGAGACCACCCACCTGCGCATCCAGGGCGAGCAGGTGCTGGAGGAGGCCCTGGAGTTC
TCCCGCAAGCAGCTGGGCGACCTGCTGGCCGAGCTGAGCTCCCCGCTGGCCGAGTACGTCAACAACTCGCTGGAGCTGCCGTACCACA
AGGGCATGCAGCGCCTGGAGGCCCGCCAGTACATCCCGATCTACGAGTCGTACGCCAACAAGAACGACACCCTGCTCCAGTTCGCCAA
GCTGGACTTCAACCTGCTCCAGGCCCTGCACCAGAGCGAGATCCGCGAGATCACCCGCTGGTGGAAGGACCTGGACTTCAAGGCCCGC
CTGCCGTACGCCCGCGACCGCCTGGTGGAGTGCTACTTCTGGATCTTGGGCGTCCAGTACGAGCCGCAGTACTCGATCAGCCGCGTGT
TCCTGACCAAGGTCATCAGCCTGGCCTCCGTGTTCGACGACACCTACGACATCTACGGCACCTTCGACGAGCTGAAGCTGCTGACCGA
CGCCGTCGAGCGCTGGGAGCCGGAGGCCACCGACTCGCTGCCGGGCTACATGCAGATCCTGTACGGCGCCCTGCTGAAGGTGTTCGAG
GAGTACAAGGACGAGCTGATCAACGCCGGCGGCCGCGACTACTGCCTGTACTACGCCAAGGAGGCCATGAAGGGCCTGGTCCGCTCCT
ACCACACCGAGGCCGTGTCGTTCCACACCGGCTACGTCCAGAACTTCGAGGAGTACCTGGACAACTCCGCCGTGTCGAGCGGCTACCC
GATGCTGACCGTCGAGGCCCTGATCGGCATGGGCGCCCCGTACGCCACCCGCGAGTCGCTGGACTGGGCCCTGAAGGTGCCGAAGATC
ATCAAGGCCTCCTCGGACATCTGCCGCCTGGTGGACGACCTGCGCACCTACAAGGTCGAGGAGGAGCGCGGCGACGCCCCGTCGGGCG
TGCACTGCTACATGCGCGACTACAACGTCAGCGAGGAGGAGGCCTGCACCAAGATCGAGGAGATGATCGACCTGGCCTGGAAGGCCAT
CAACGAGGAGATCCAGAAGCCGAACCACCTGCCGCTGCCGATCCTGCTGCCGGCCCTGAACTTCGCCCGCATGATGGAGGTGCTGTAC
CAGAACATCGACGGCTACACCAACAGCGGCGGCCGCACCAAGGAGCGCATCAGCTCCCTGCTGGTCCACCCGTTCACCATCTGA

A

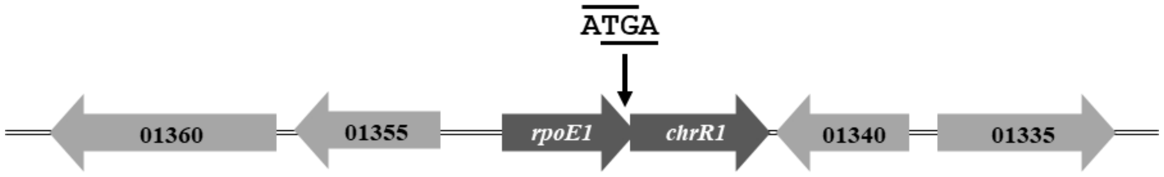

B

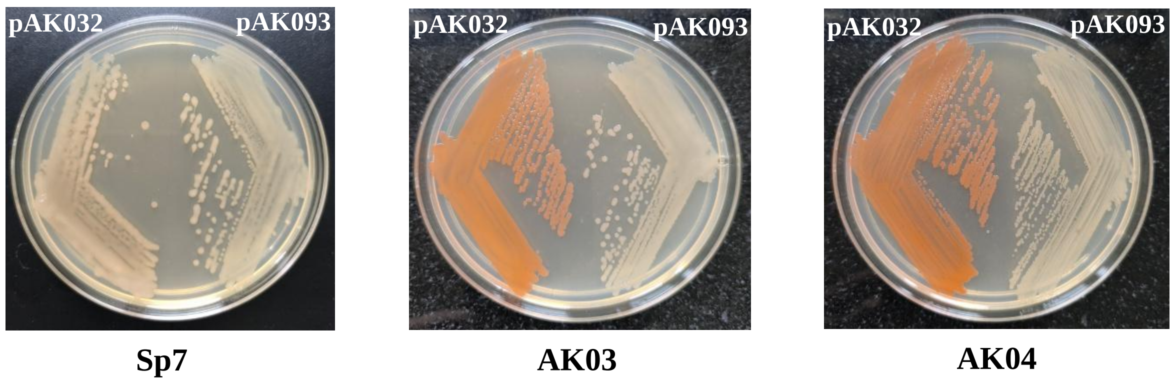

Suppl. Figure 1. (A) Schematic presentation of the *rpoE1-chrR1* loci in *Azospirillum brasilense* Sp7. Horizontal thick arrows represent the relative positions and transcriptional orientations of the ORFs; AMK58_01360 (23S rRNA methyltransferase), AMK58_01355 (hypothetical protein), *rpoE1*, *chrR1*, AMK58_01340 (rRNA methyltransferase) and AMK58_01335 (L-asparaginase II). Nucleotides shared by *rpoE1* and *chrR1* ORFs are shown with vertical arrows; *rpoE1* stop codon is underlined while *chrR1* start codon is overlined. **(B)** Complementation of AK03 and AK04 mutants. Luria agar plates showing colonies of Sp7, AK03 and AK04 harbouring empty vector (pAK032) or *chrR1*-expressig plasmid (pAK093).

1	3 Malate + 3 NAD	Malate dehydrogenase →	3 Oxaloacetate + 3 NADH
2	3 Oxaloacetate + 3 ATP	PEP Carboxykinase →	3 PEP + 3 CO_2 + 3 ADP
3	PEP + ATP + NADH	Gluconeogenic route →	G3P + ADP + NAD
4	2 PEP + 2 ADP	Pyruvate kinase →	2 Pyruvate + 2ATP
5	Pyruvate + NAD	Pyruvate dehydrogenase →	Acetyl-CoA + NADH + CO_2
6	Acetyl-CoA + 3 NAD + FAD + ADP	TCA cycle →	3 NADH + FADH + ATP + 2 CO_2
7	3 NADH + 7.5 ADP	Electron transport system →	7.5 ATP + 3 NAD
8	3 Malate + 3 NAD + 6.5 ADP + FAD	Eq. 1 - 7 →	G3P + Pyruvate + 3 NADH + 6.5 ATP + FADH + 6 CO_2
9	G3P + Pyruvate + 3 NADPH + 2 ATP	DXP Pathway →	IPP + CO_2 + 3 NADP + 2 ADP
10	3 Malate + 6 ADP + FAD	Eq. 8 - 9 →	IPP + 4.5 ATP + FADH + 7 CO_2

Suppl. Figure 2. Stoichiometries of the glycolytic, gluconeogenic, TCA cycle and DXP pathway reactions involved in conversion of malate to IPP/DMAPP. Total 3 malate molecules are required for one molecule of IPP or DMAPP: two for biosynthesis of the DXP pathway starting substrates (pyruvate and glyceraldehyde 3-phosphate (G3P)) and one for production of the required NADPH and ATP. NADH and NADPH are considered equally in the calculations due to their inter-convertible nature. Since activity of malic enzyme(s) (enzyme 2 in panel A) are not known in *A. brasilense*, reaction catalysed by this enzyme has not been included in the calculations; however, the malic enzyme-mediated utilization will give the same outcome with consumption of an additional ATP/malate molecule.

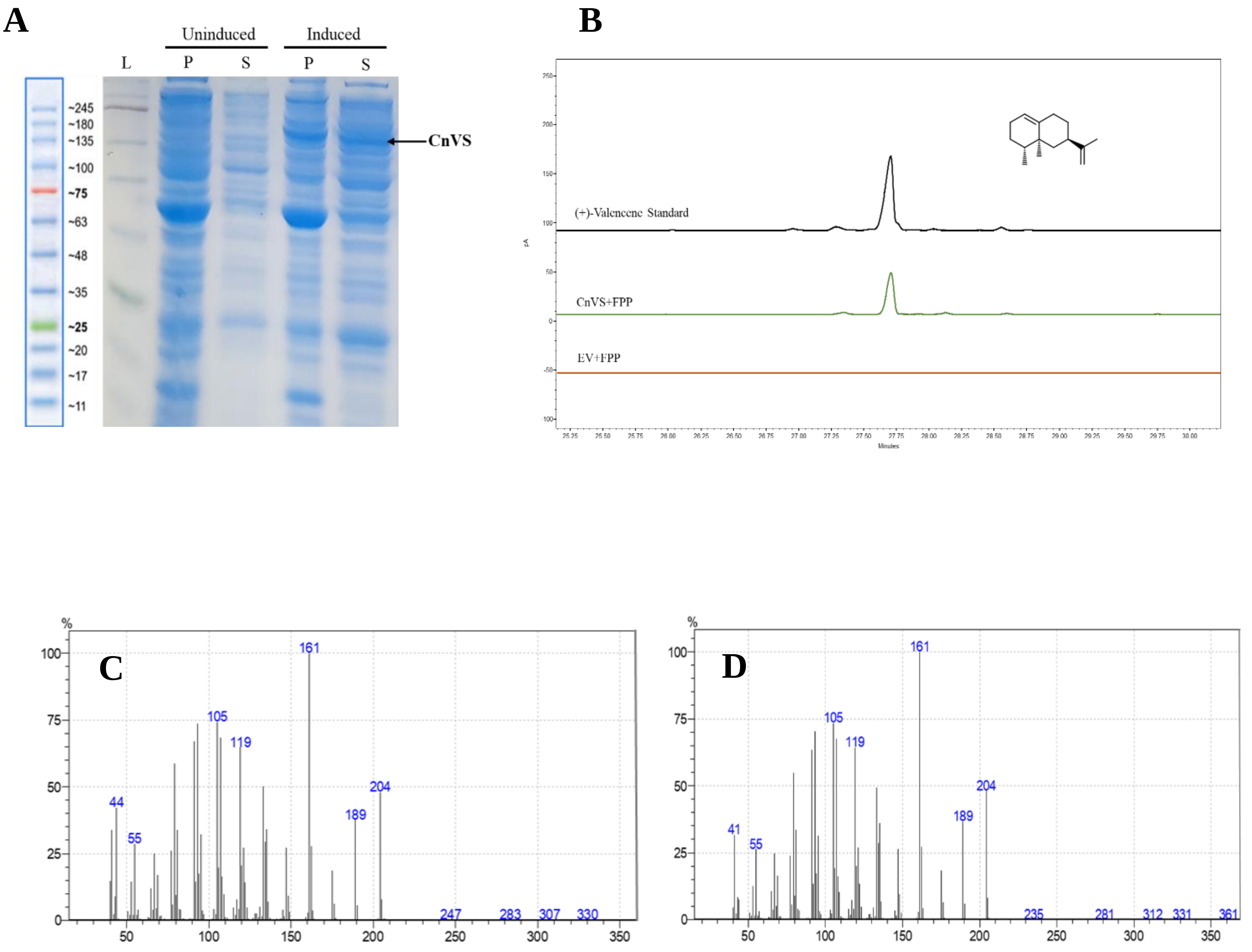

Suppl. Figure 3. (A) SDS-PAGE analysis of the proteins extracted from soluble (S) and pellet (P) fractions prepared from uninduced and induced cultures of *E. coli* BL21(DE3) pLysS cells horbouring pAK052 (expressing CnVS). **(B)** GC analysis of hexane extracts prepared from the *in vitro* enzymatic reactions performed with the soluble fractions of *E. coli* BL21(DE3) pLysS cells harbouring pAK052 (expressing CnVS) or pAK032 (empty vector). Mass spectra of the (+) valencene standard (**C**) and the corresponding peak achieved with the CnVS (**D**).

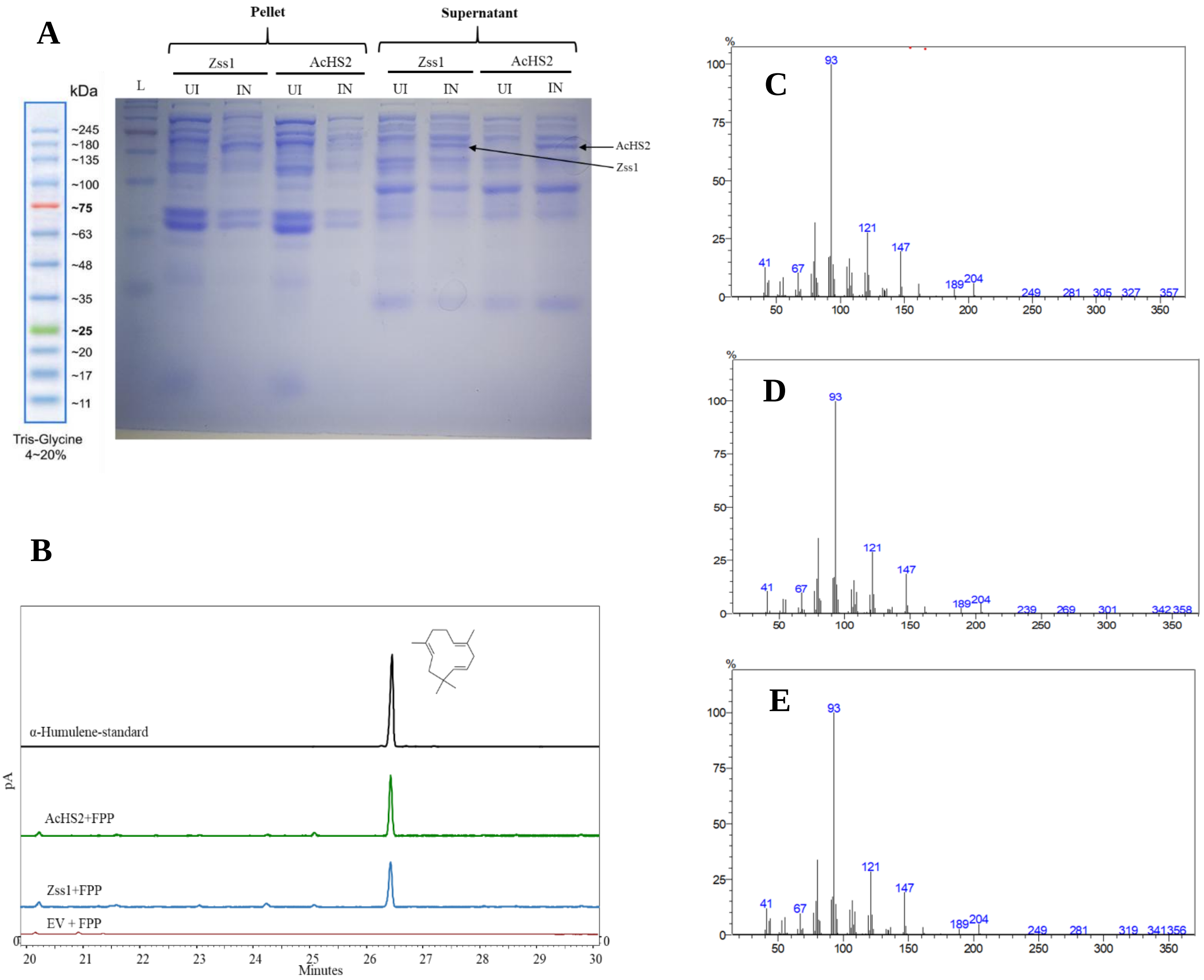

Suppl. Figure 4. (A) SDS-PAGE analysis of the soluble (supernatant) and pellet fractions prepared from uninduced (UN) and induced (IN) cultures of *E. coli* BL21(DE3) pLysS cells harbouring pAK053 (expressing ZSS1) or pAK060 (expressing AcHS2). **(B)** GC analysis of hexane extracts prepared from the *in vitro* enzymatic reactions performed with the soluble fractions of *E. coli* BL21(DE3) pLysS cells horbouring pAK053 (expressing Zss1), pAK070 (expressing AcHS2) or empty vector. Mass spectra of the α-humulene standard (**C**) and the corresponding peaks achieved with ZSS1 (**D**) and AcHS2 (**E**).

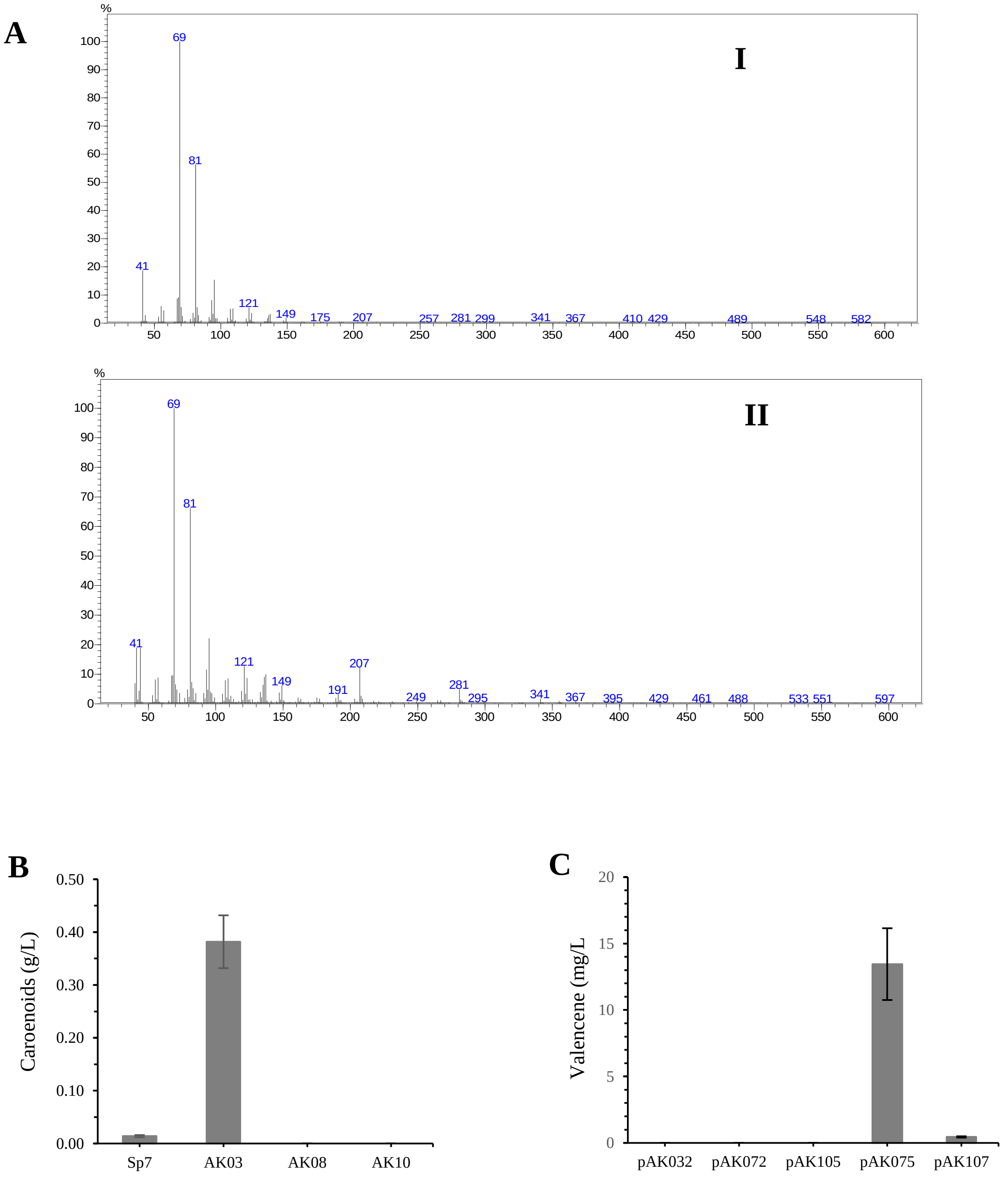

Suppl. Figure 5. **(A)** Mass-spectra of squalene standard (I) and the corresponding peak detected in the organic extract prepared from AK10 harbouring pAK092 (pAK032-*rpoE1*) (II). **(B)** Comparison of carotenoid contents (g/L) in Sp7 (parent), AK03 (*chrR1*::Km), AK08 (*ΔhpnCDE*) and AK10 (*ΔcrtNPOQ*). **(C)** Comparison of valencene yields by AK08 strains harbouring pAK032 (empty vector), pAK072 (pAK032-*CnVS*), pAK105 (pAK032-*CnVS-rpoE1*), pAK075 (pAK032-*CnVS-ispA-dxs-idi*) and pAK107 (pAK032-*CnVS-ispA-dxs-idi-rpoE1*). Each bar shows the mean and standard deviation of values obtained from three replicates.

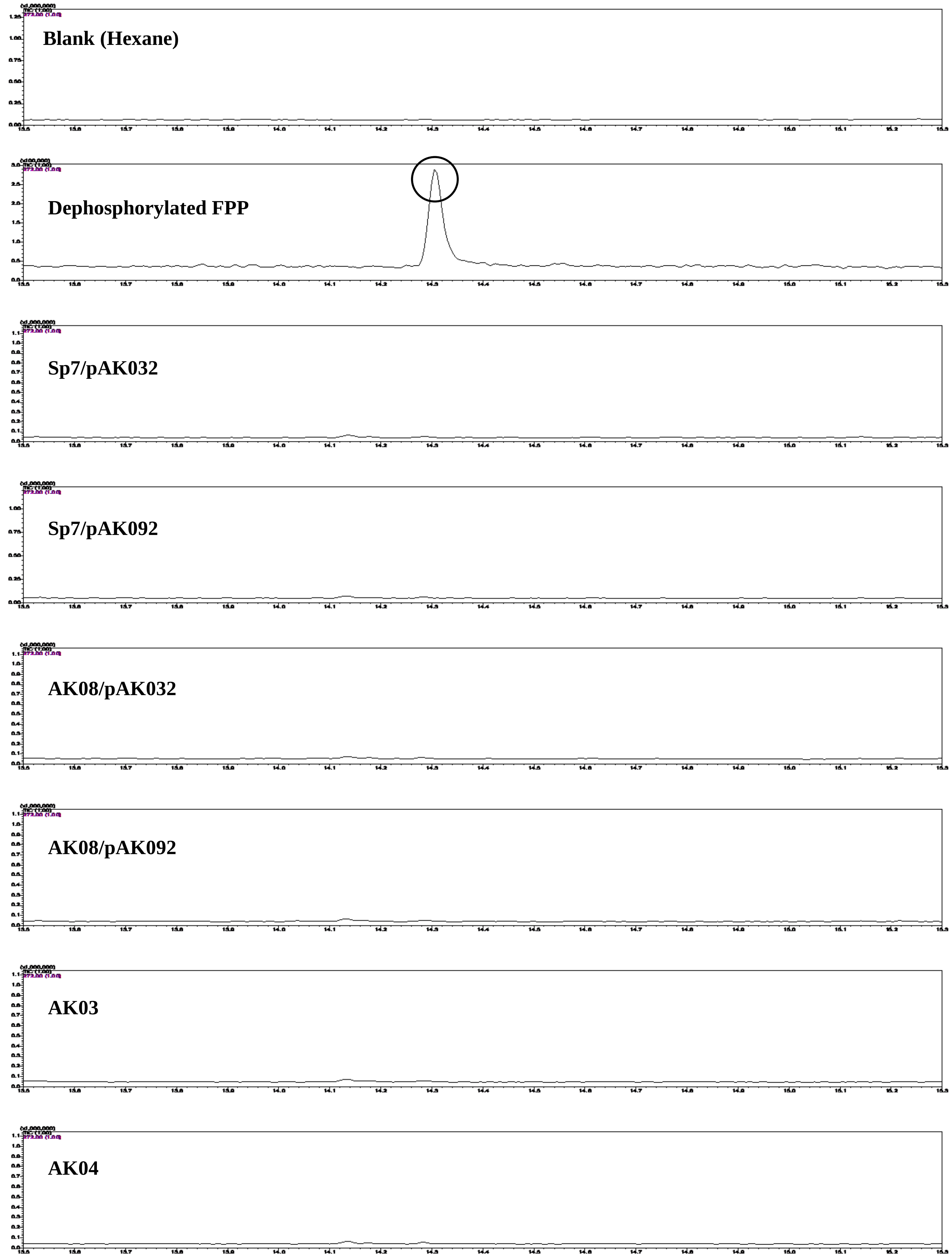

Suppl. Figure 6. GC-MS chromatograms of hexane extracts of the dephosphorylation reactions performed to convert the FPP (authentic standard or extracted from different *Azospirillum brasilense* Sp7 strains) into farnesol. The farnesol-specific peak has been encircled. The x axis denotes the retention time in minutes and the y axis denotes peak intensity.

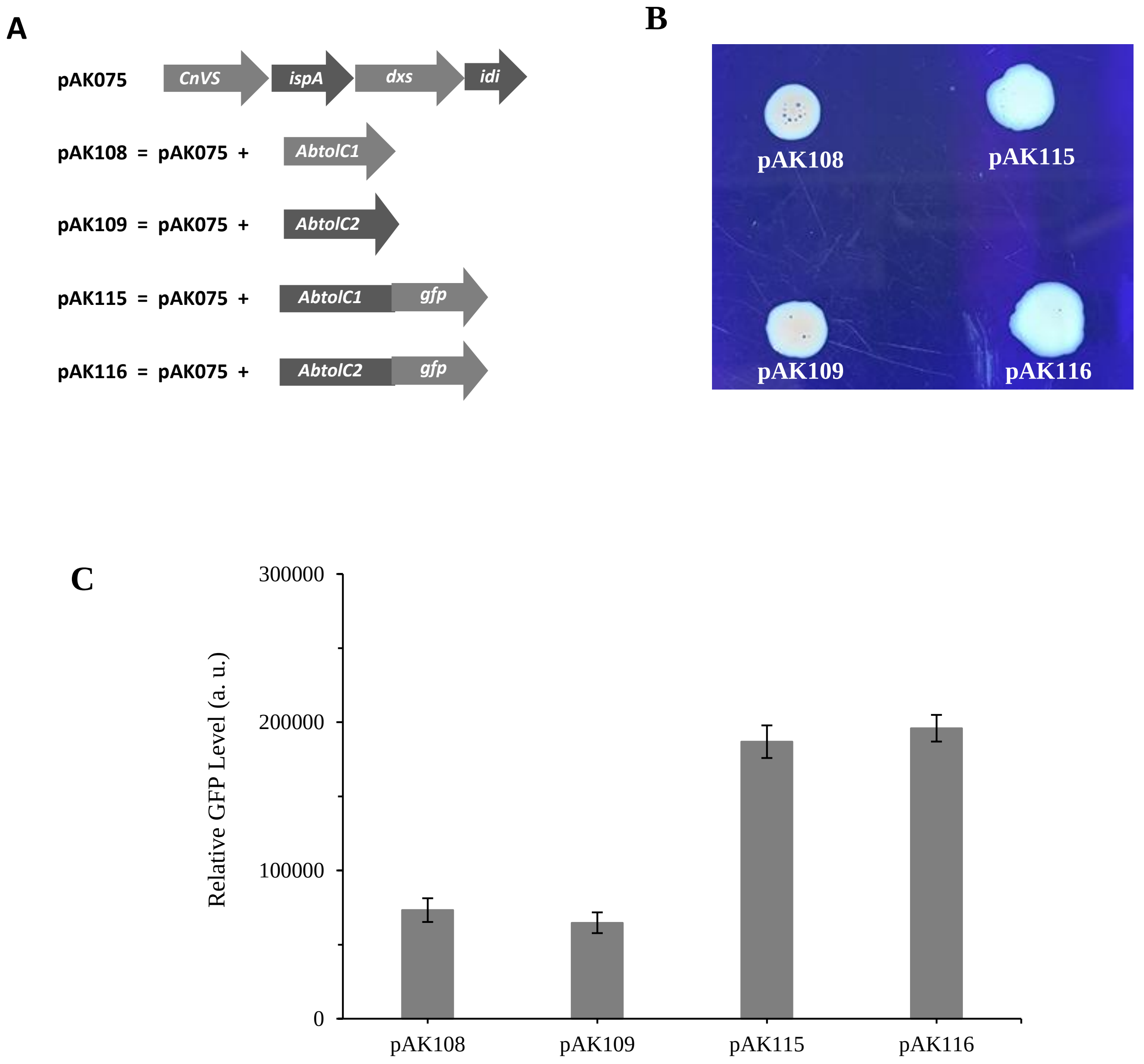

Suppl. Figure 7. (A) Schematic presentation of the genes presents in different plasmids constructed by insertion of *AbtolC1* and *AbtolC2* (genes encoding outer membrane proteins) and their fused ORFs with GFP in pAK075. **(B)** Qualitative analysis of the GFP level in AK03 cells harbouring different plasmids by visual inspection of the cell pellets under UV background. **(C)** Quantitative comparison of GFP level in AK03 cells harbouring different plasmids using fluorometric analysis. For both the analyses, equal volume of the cultures was used after adjusting their optical density.
